# Supplementary material for: Bioelectric stimulation controls tissue shape and size
Source: Nat Commun. 2024 Apr 5;15:2938. doi: 10.1038/s41467-024-47079-w (PMC10997591; doi:10.1038/s41467-024-47079-w)
Supplement: Supplementary file 1 — Supplementary Information [file 41467_2024_47079_MOESM1_ESM.pdf]

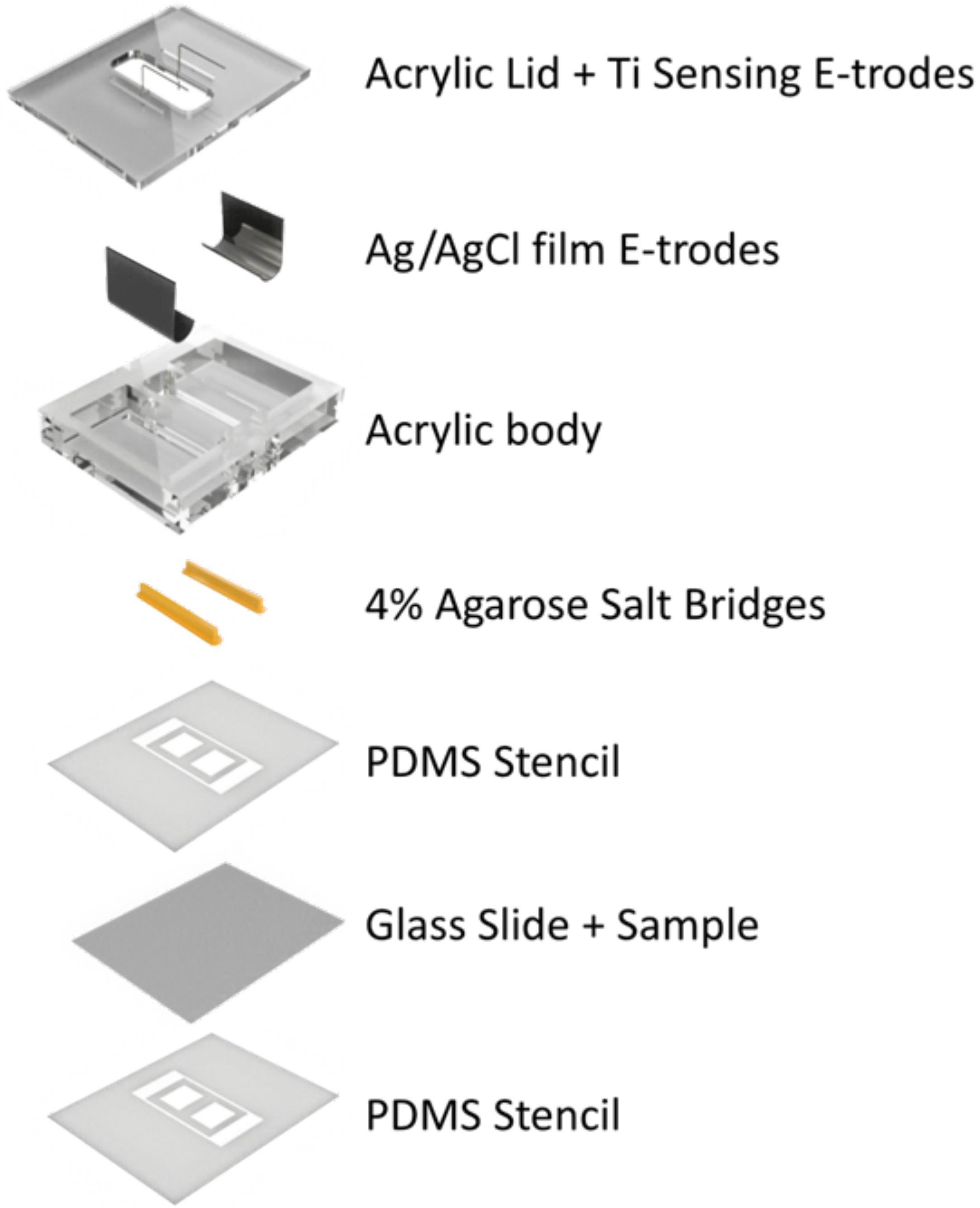

Supplementary Figure 1 Individual components of the electric bioreactor setup. Silver/silver-chloride electrodes connected directly to a current supply were submerged into a PBS reservoir on either side of the PDMS microfluidics chamber to deliver electric field through agarose salt bridges.

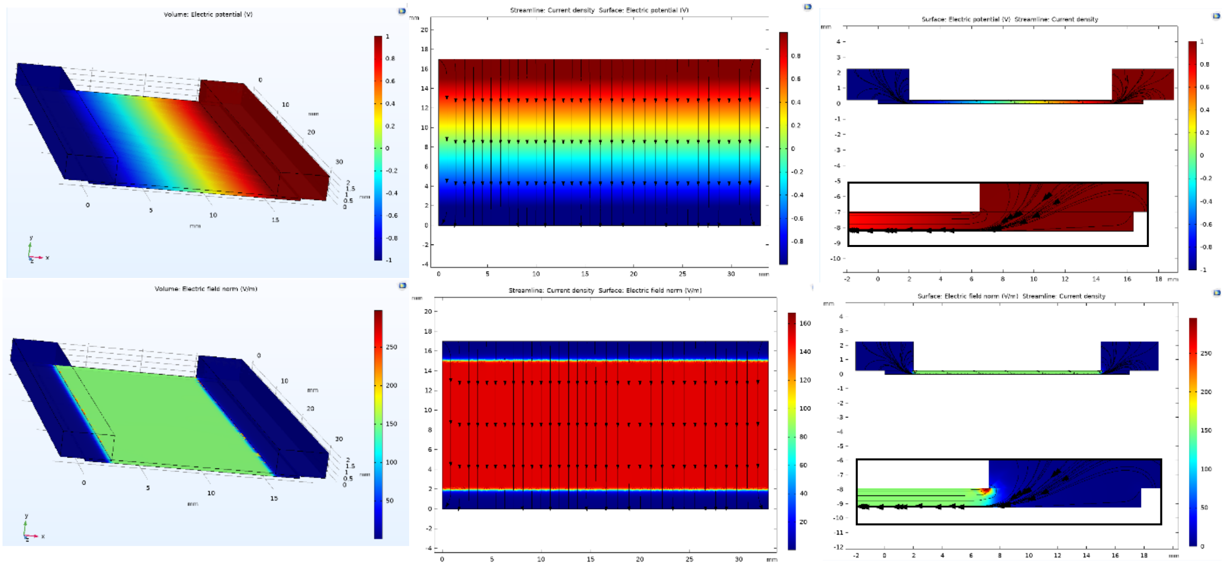

Supplementary Figure 2 Left: 3D Finite Element simulation of Electric Potential (*Top*) and Field Strength (*Bottom*) in the bioreactor used for Cyst stimulation. **Middle**: Top-down view of current density (black streamlines) within the microfluidic channel where cysts are cultured and stimulated. **Right**: Cross-sectional side view of current density (black streamlines) within the microfluidic channel where cysts are cultured and stimulated. (**Inset**) Current density at the salt bridge-electrolyte interface shows slight fringing far from the region of the device containing the cysts.

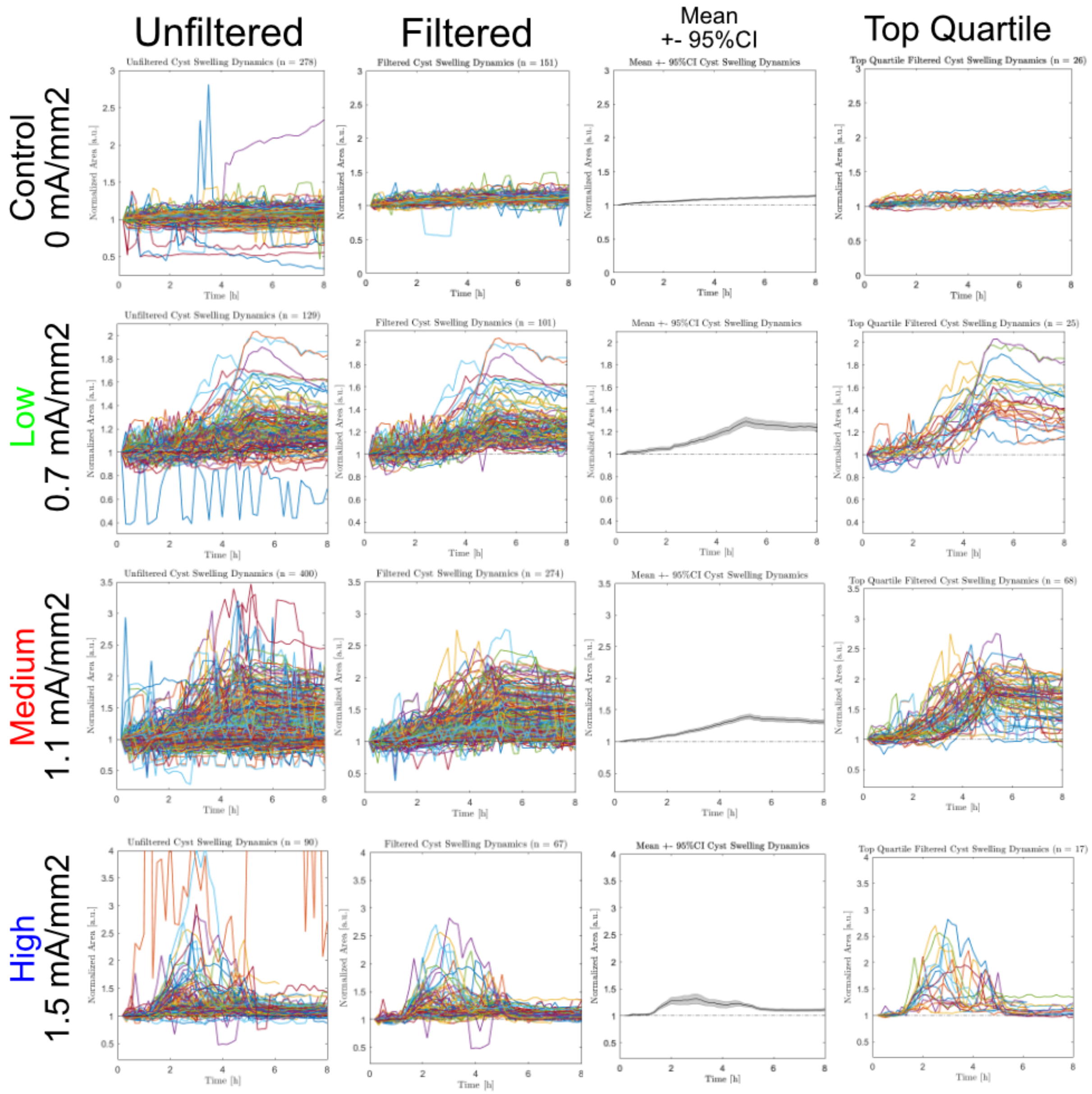

Supplementary Figure 3 Raw, filtered, averaged, and top quartile (left to right) plots for control, low, medium, and high stimulation (top to bottom). For all experiments, all cysts were binarized to produce raw area plots throughout the duration of the experiment. The raw cyst area plots were filtered for irregularities that arise from image processing, then averaged and plotted alongside the 95% confidence interval. The top quartile was calculated to produce the final plot.

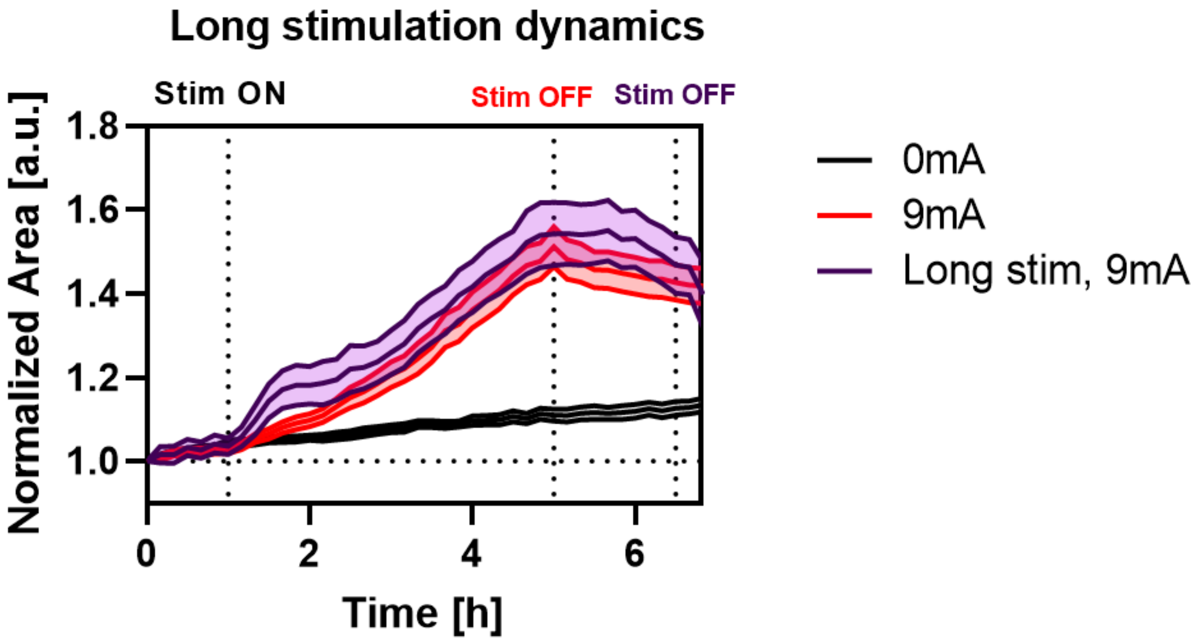

Supplementary Figure 4 Comparison between MDCK acini stimulated at medium current for 4h (red; stimulation on from 1h – 5h; N = 150) and for 5.5h (purple; stimulation on from 1h – 6.5h; N = 1, n = 72). Longer stimulation does not increase the cyst volume further, suggesting that the maximum inflation magnitude under electrical stimulation is capped for MDCK cysts.

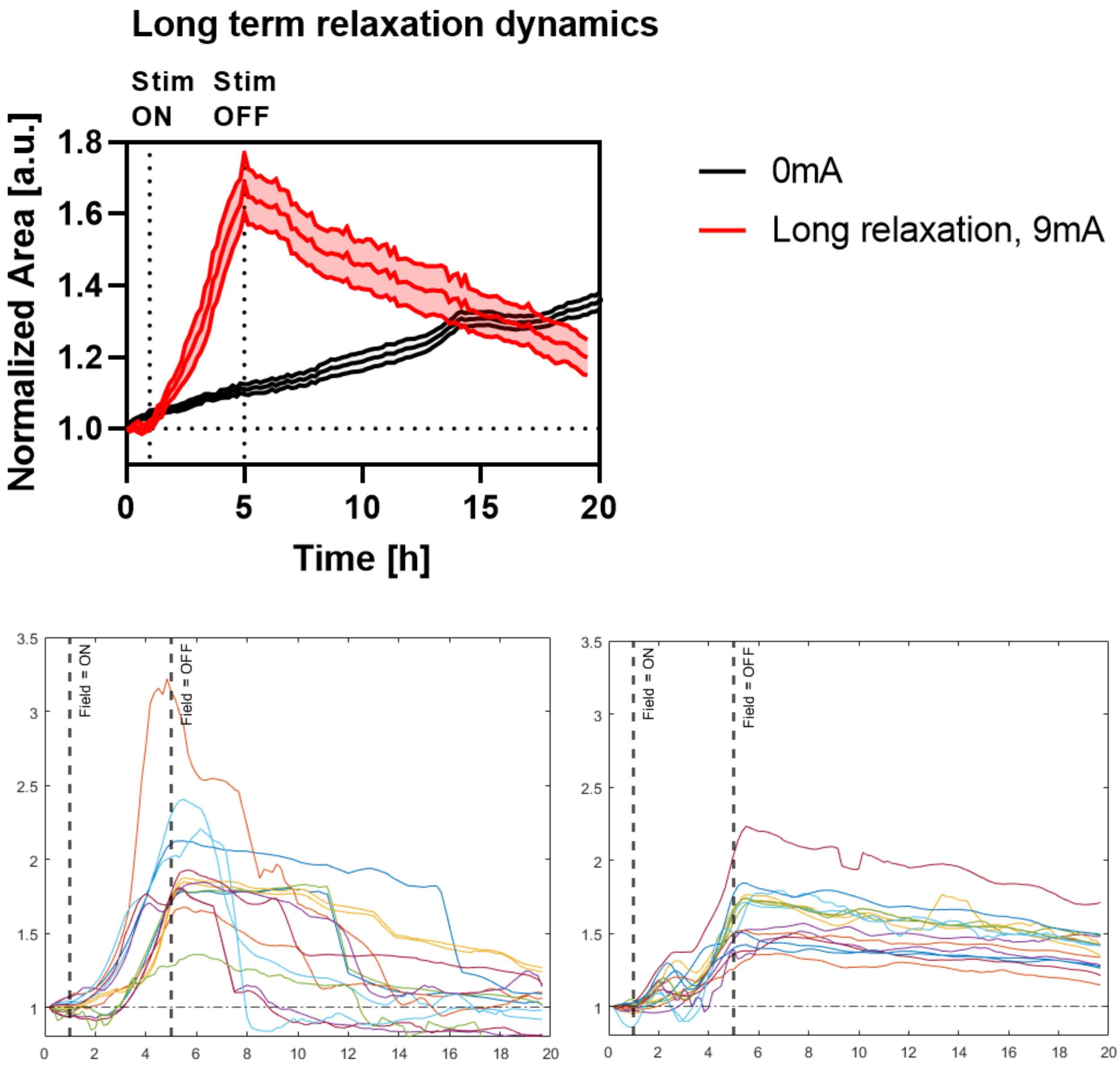

Supplementary Figure 5 Normalized area as a function of time capturing deflation dynamics of MDCK acini electrically stimulated at medium current stimulation. Stimulation is applied at the 1h mark, maintained for 4h, then removed (N = 1, n = 80). Deflation is slow and gradual. Area dynamics during long relaxation is bimodal. **Top**: Averaged normalized area plot of all cysts electrically stimulated then relaxed (green) and cysts with no electrical stimulation (grey) **Bottom left:** Individual cysts' normalized area plots with rapid deflation, where relaxing cysts suddenly drop in volume. **Bottom right:** Individual cysts' normalized area plots with steady deflation, where they drop in volume slowly.

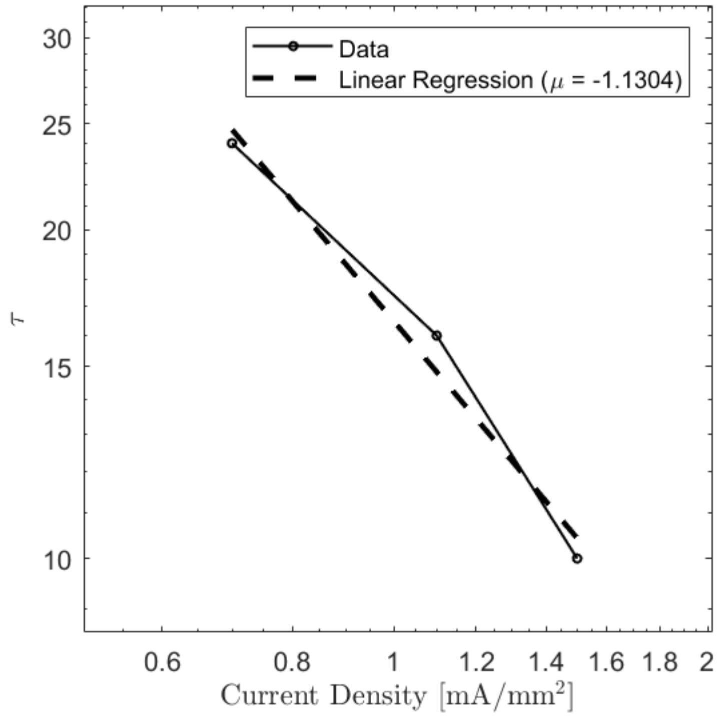

Supplementary Figure 6 Linear regression of threshold time T as a function of current density, plotted in log-log form. T is defined to be the time at which the normalized area curve for each current density reaches some arbitrary threshold normalized area. The slope of the fit is then used to rescale the normalized area curves onto a single master curve (see Fig. 1C).

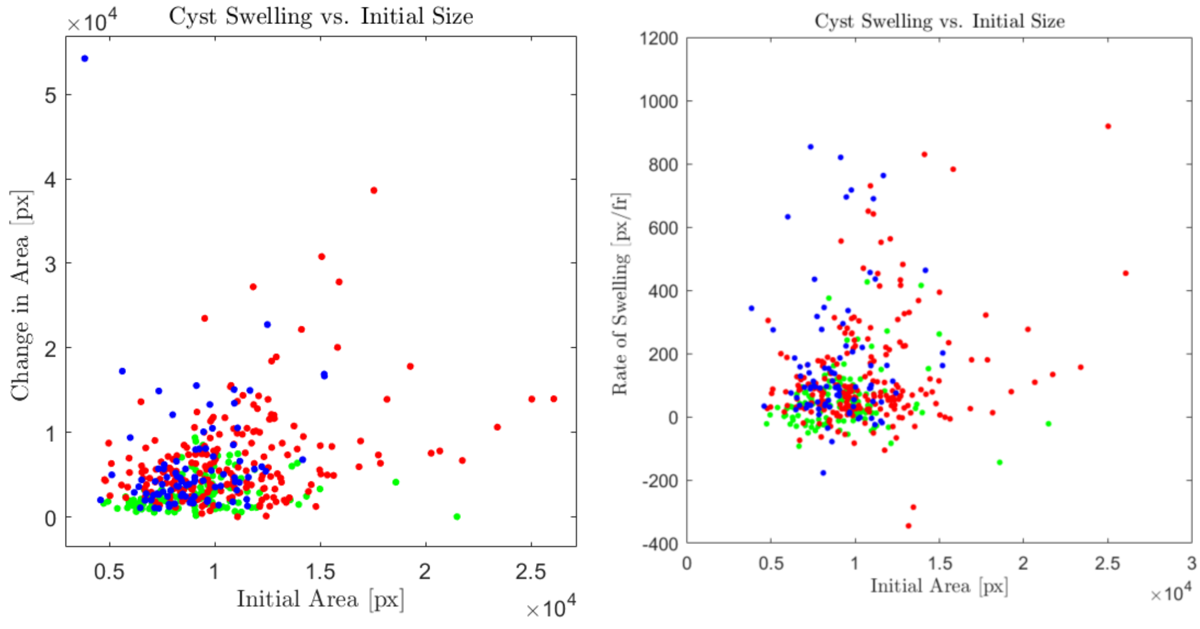

Supplementary Figure 7 Left: Maximum normalized area vs. initial area of each cyst after 3 hours of stimulation at low (green), medium (red), and high (blue) current. Initial size has little to no effect on the final size of the cyst. **Right:** Mean rate of swelling in the first hour of stimulation for all cysts across low (green), medium (red), and high (blue) current. Initial cyst area does not affect the rate of swelling within each experimental condition.

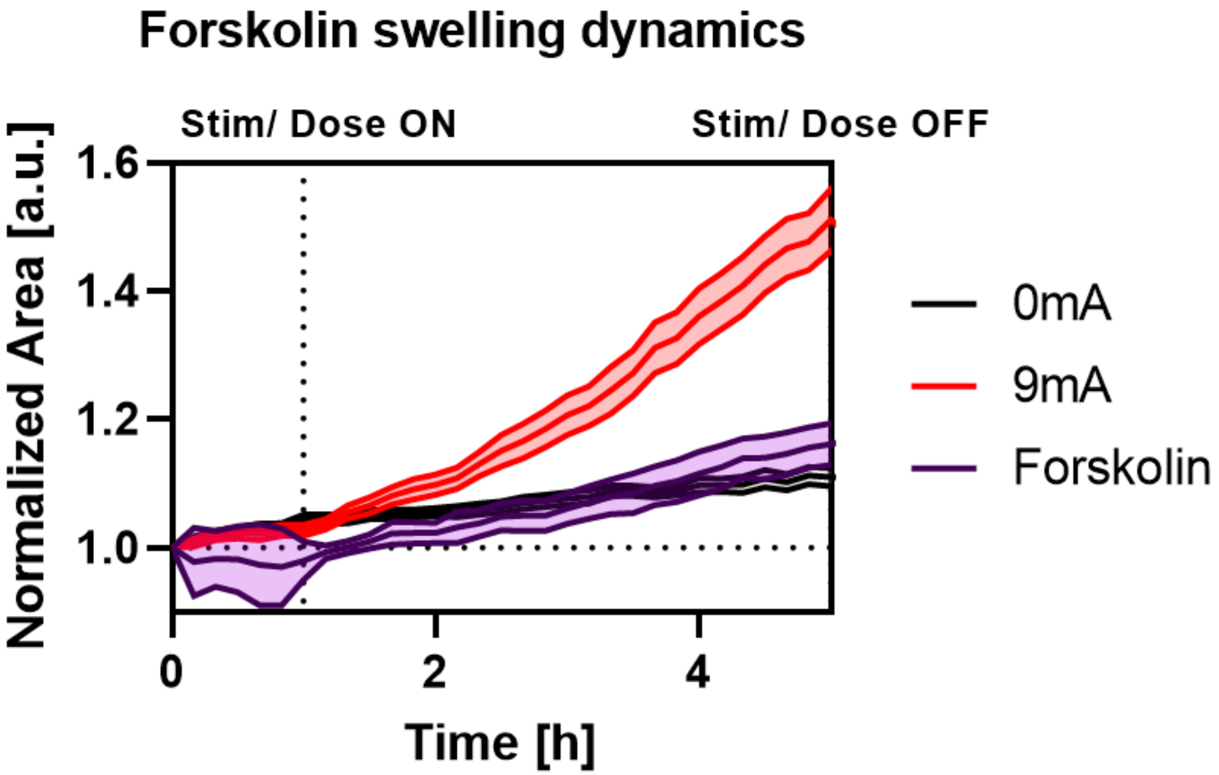

Supplementary Figure 8 Comparison between 100uM forskolin treatment (purple; N = 1, n = 30) and electrical stimulation at medium current (red; N = 5, n = 150). Electrical stimulation and chemical stimulation is applied at the 1h mark and maintained for 4h.

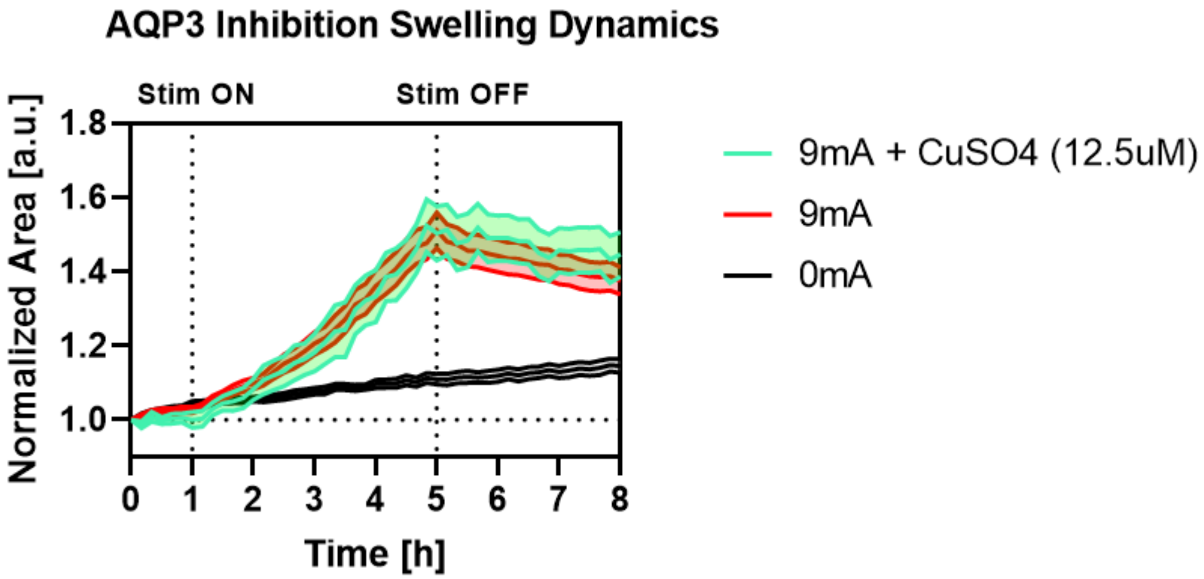

Supplementary Figure 9 Comparison between MDCK acini without stimulation nor treatment (black; N = 5, n = 145), stimulated with no treatment for 4h (red; N = 5, n = 150), and stimulated with CuSO4 treatment for aquaporin 3 (AQP3) inhibition (teel; N = 3, n = 101). There is no significant reduction in cyst inflation with the inhibition of AQP3.

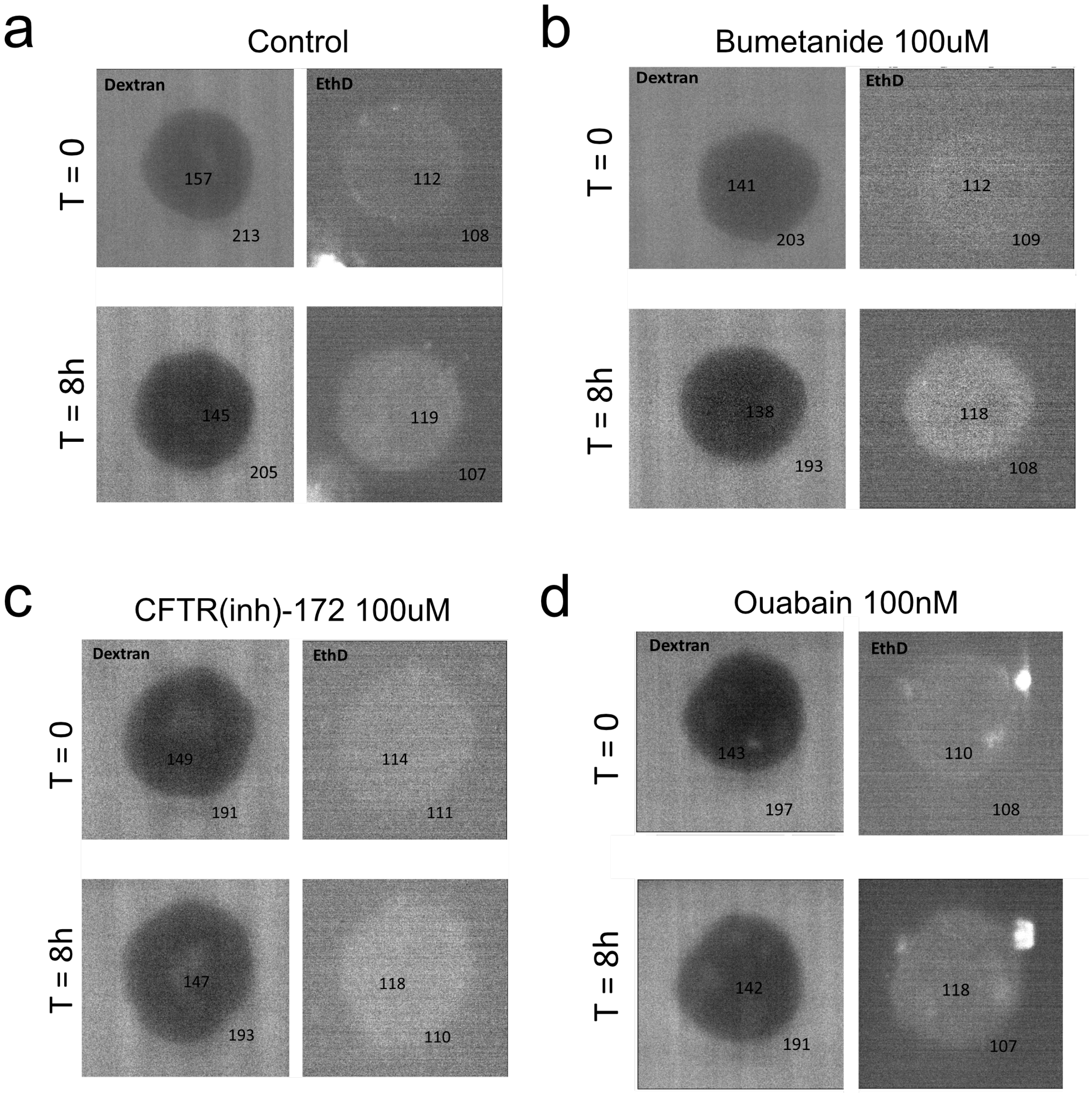

Supplementary Figure 10: Dextran and EthD-1 assay to test epithelial barrier function and cytotoxicity with the addition of inhibitors. Inhibitors were added 24h (CFTR(inh)-172, ouabain) or 1h (bumentanide) before the experiment. The fluorescence values within the lumen and external environment are overlayed on the image. In all conditions, the 4kDa dextran did not penetrate the lumen and there was no nuclear localization of EthD-1, confirming that the inhibitor treatment did not induce the concerned effect to our model systems.

	Cytoskeletal Inhibitors					
	Control	1.1 mA/mm²	Y27632 + 1.1 mA/mm²	Y27632	Blebbistatin + 1.1 mA/mm²	Blebbistatin
**Control**	1					
**1.1 mA/mm²**	<0.0001 (****)	1				
**Y27632 + 1.1 mA/mm²**	<0.0001 (****)	<0.0001 (****)	1			
**Y27632**	<0.0001 (****)	<0.0001 (****)	<0.0001 (****)	1		
**Blebbistatin + 1.1 mA/mm²**	<0.0001 (****)	<0.0001 (****)	0.7247 (n.s)	<0.0001 (****)	1	
**Blebbistatin**	<0.0001 (****)	<0.0001 (****)	<0.0001 (****)	0.0087 (**)	<0.0001 (****)	1

	Ion Channel Inhibitors									
	Control	1.1 mA/mm²	CFTR-172 50uM + 1.1 mA/mm²	CFTR-172 100uM + 1.1 mA/mm²	CFTR-172 100uM	Bumetanide 100uM + 1.1 mA/mm²	Bumetanide 100uM	Ouabain 100 nM	Ouabain 100 nM + 1.1 mA/mm²	
Control	1									n.s.
1.1 mA/mm²	<0.0001 (****)	1								(*)
CFTR-172 50uM + 1.1 mA/mm²	<0.0001 (****)	<0.0001 (****)	1							(**)
CFTR-172 100uM + 1.1 mA/mm²	<0.0001 (****)	<0.0001 (****)	<0.0001 (****)	1						(***)
CFTR-172 100uM	<0.0001 (****)	<0.0001 (****)	<0.0001 (****)	<0.0001 (****)	1					(****)
Bumetanide 100uM + 1.1 mA/mm²	<0.0001 (****)	0.0127 (*)	<0.0001 (****)	<0.0001 (****)	<0.0001 (****)	1				
Bumetanide 100uM	0.0036 (**)	<0.0001 (****)	<0.0001 (****)	<0.0001 (****)	<0.0001 (****)	<0.0001 (****)	1			
Ouabain 100 nM	0.0433 (*)	<0.0001 (****)	<0.0001 (****)	<0.0001 (****)	0.0172 (*)	<0.0001 (****)	0.0144 (*)	1		
Ouabain 100 nM + 1.1 mA/mm²	<0.0001 (****)	0.0201 (*)	0.8205 (n.s.)	<0.0001 (****)	<0.0001 (****)	0.0689 (n.s.)	<0.0001 (****)	<0.0001 (****)	1	

Supplementary Figure 11: Lookup table of p-values for all experimental conditions. Colors represent significance as measured by a pairwise parametric t-test with Welch's correction. Data distributions were verified to be gaussian by fitting using MATLAB.

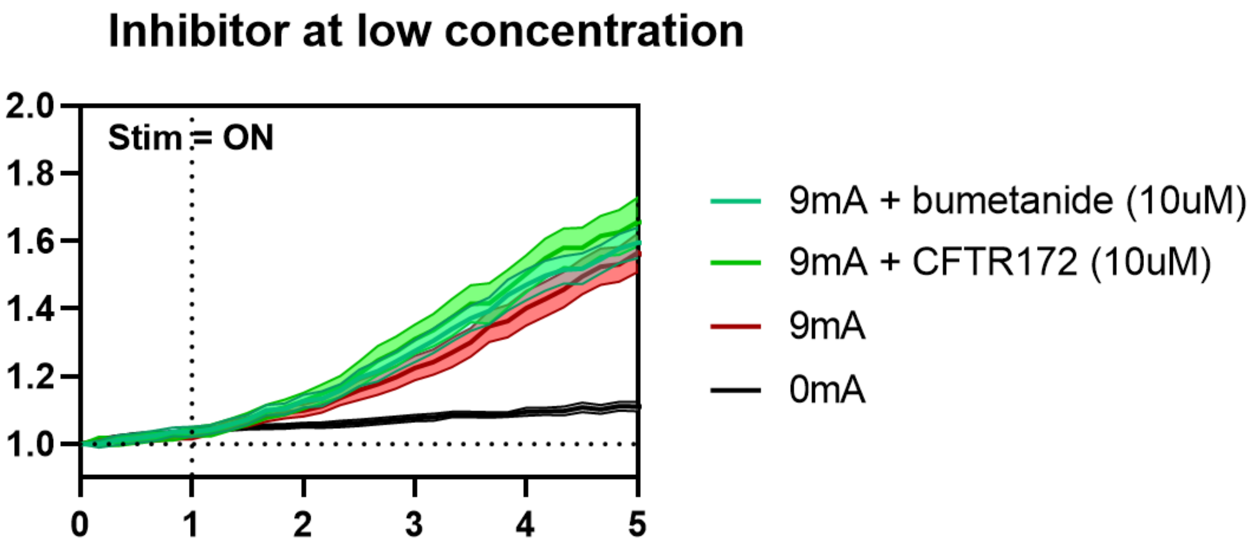

Supplementary Figure 12: Swelling dynamics for lower concentration of inhibitors. CFTR(inh)-172, 10uM (green; N = 2, n = 78) and bumetanide 10uM (teel; N = 3, n = 150). There were no significant differences in inflation in cysts treated with lower concentration of inhibitors.

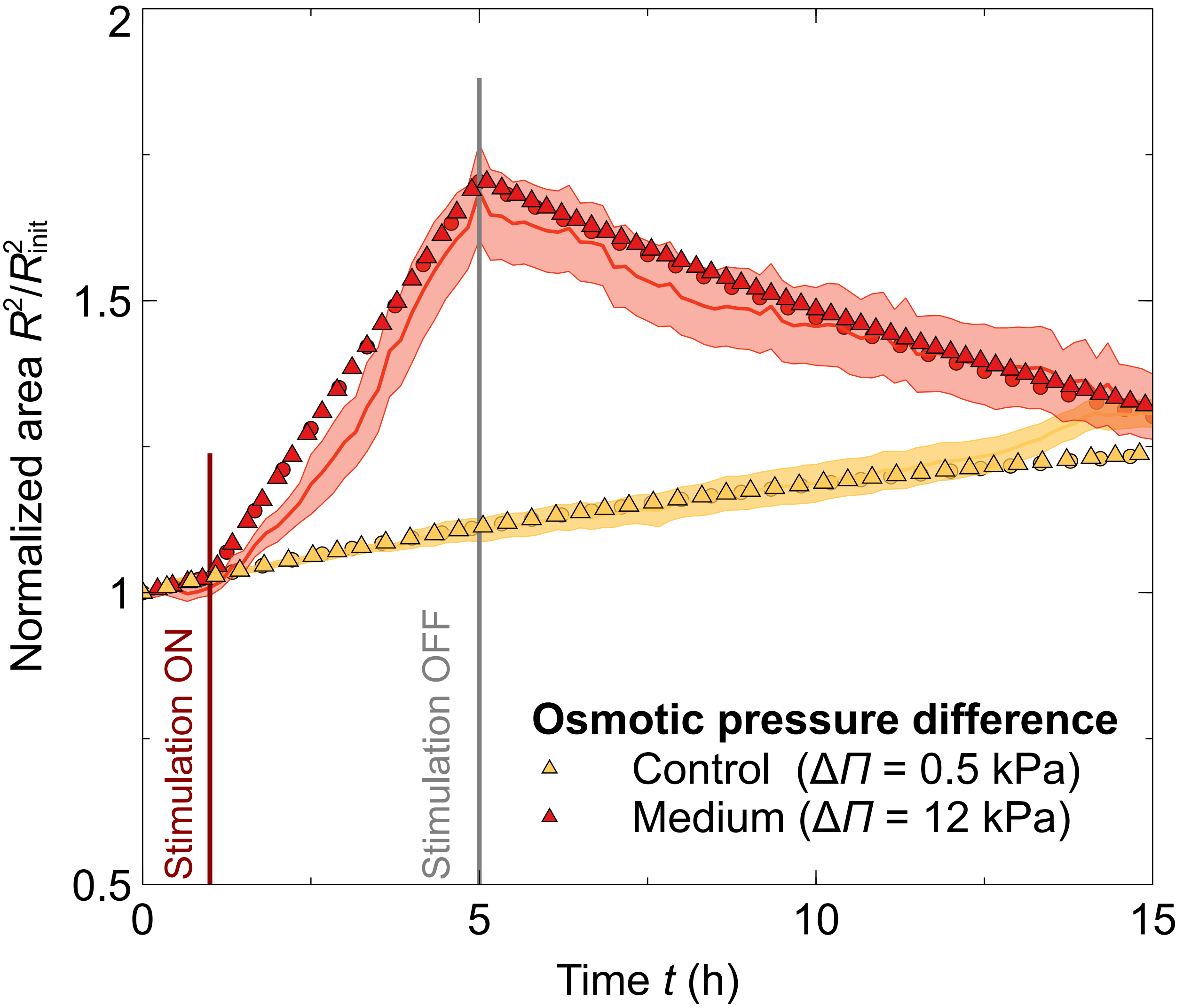

Supplementary Figure 13 Normalized area as a function of time obtained from experiments (curves) and 1D model (triangles) considering a Maxwell material. In the model, we consider that the osmotic pressure increases drastically due to stimulation but remains constant over time. When the field is turned off, the osmotic pressure is decreased drastically, e.g., due to ion rearrangement or fluid leakage. The build-up hydrostatic normal stress overcomes the osmotic pressure, thus driving cyst shrinkage. See Supplementary Information for further details.

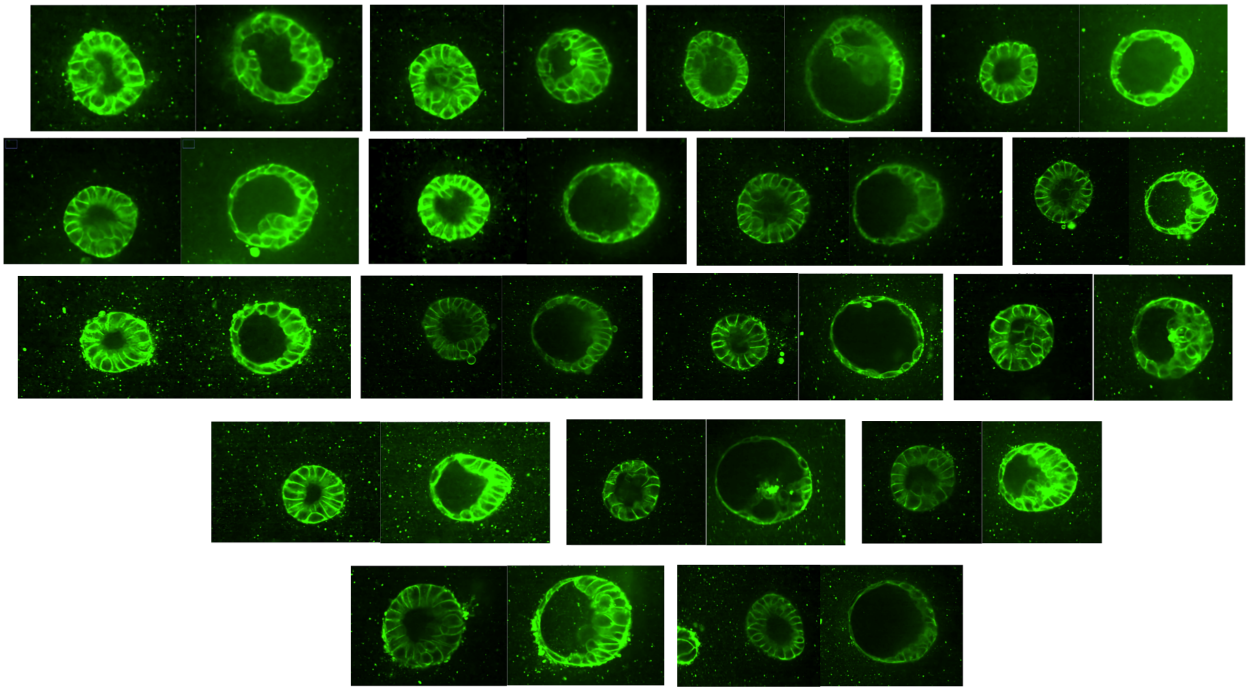

Supplementary Figure 14 The heterotypic response of electrically stimulated cysts. Some cysts undergo significant areal increase with a radially symmetric response, whereas some cysts do not swell as much, and will instead thicken on the cathode-facing half and thin on the anode-facing half.

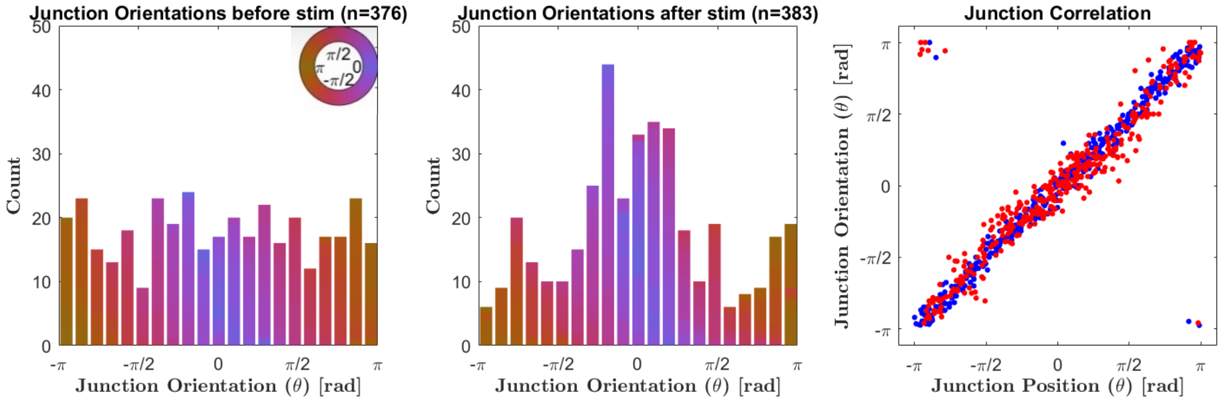

Supplementary Figure 15 Left: Uniform distribution of junction orientations before stimulation. **Middle**: Distribution of junction orientations after stimulation shows that junctions on the cathode-facing half of the cyst tend to be more horizontally oriented. **Right**: Scatter plot of junction orientation vs. junction position. Before (blue) and after (red) stimulation, there is a linear relationship between position and orientation.

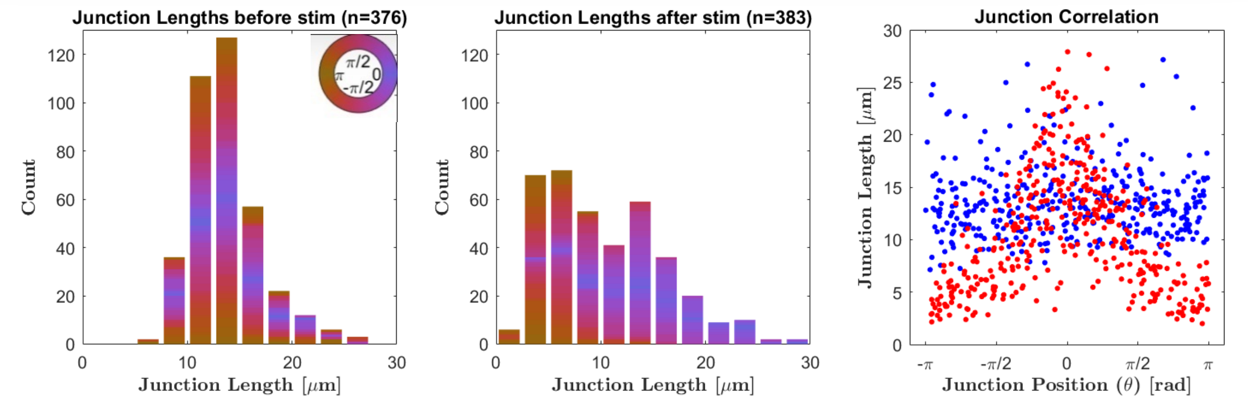

Supplementary Figure 16 Left: Distribution of junction lengths before stimulation showing that junctions throughout the perimeter of the cyst tend to have a mean length of ~15um. **Middle:** Distribution of junction lengths after stimulation showing that junctions on the anode-facing half of the cyst tend to be shorter than the original mean length of ~15um whereas junctions on the cathode-facing half of the cyst tend to be longer than ~15um. **Right**: Scatter plot of junction length vs. junction position. Before (blue) stimulation, there is no relationship between junction length and position in the cyst circumference, whereas after (red) stimulation, junctions tend to be longer on the cathode-facing half of the cyst ($-\pi/2 < \theta < \pi/2$).

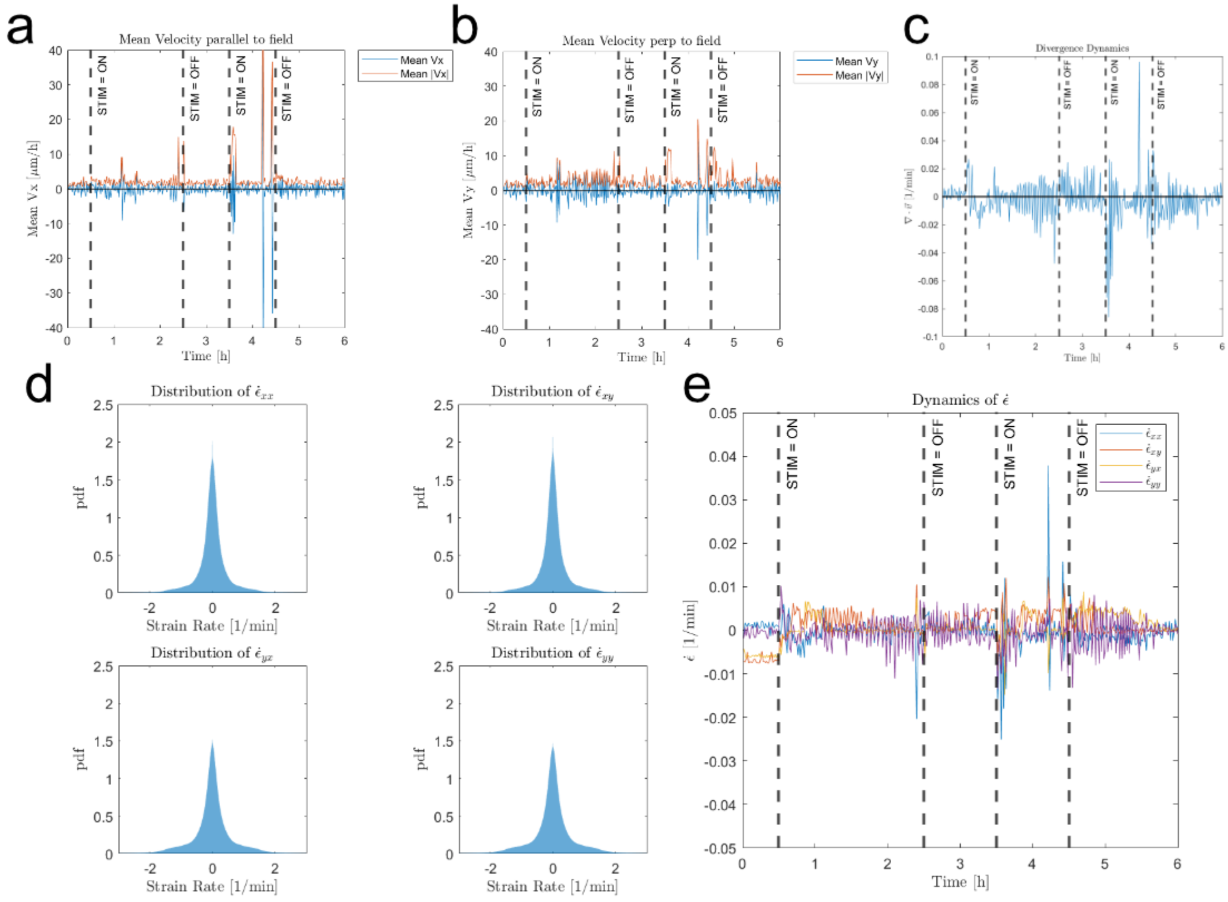

Supplementary Figure 17 Several deformation analyses were performed on electrically stimulated hydrogels that did not contain any MDCK cysts. (a-b) Mean velocity and speed (a) parallel and (b) perpendicular to the field showed no significant change during stimulation periods. (c) Divergence of the velocity field in the hydrogel remained negligible with and without stimulation. (d) Probability density functions of all entries in the strain rate tensor in the hydrogel during stimulation. Distributions are centered at and symmetrical about zero, suggesting that the gel is not undergoing any directed stress. (e) Temporal dynamics of mean strain rate remain zero during all times, with and without stimulation.

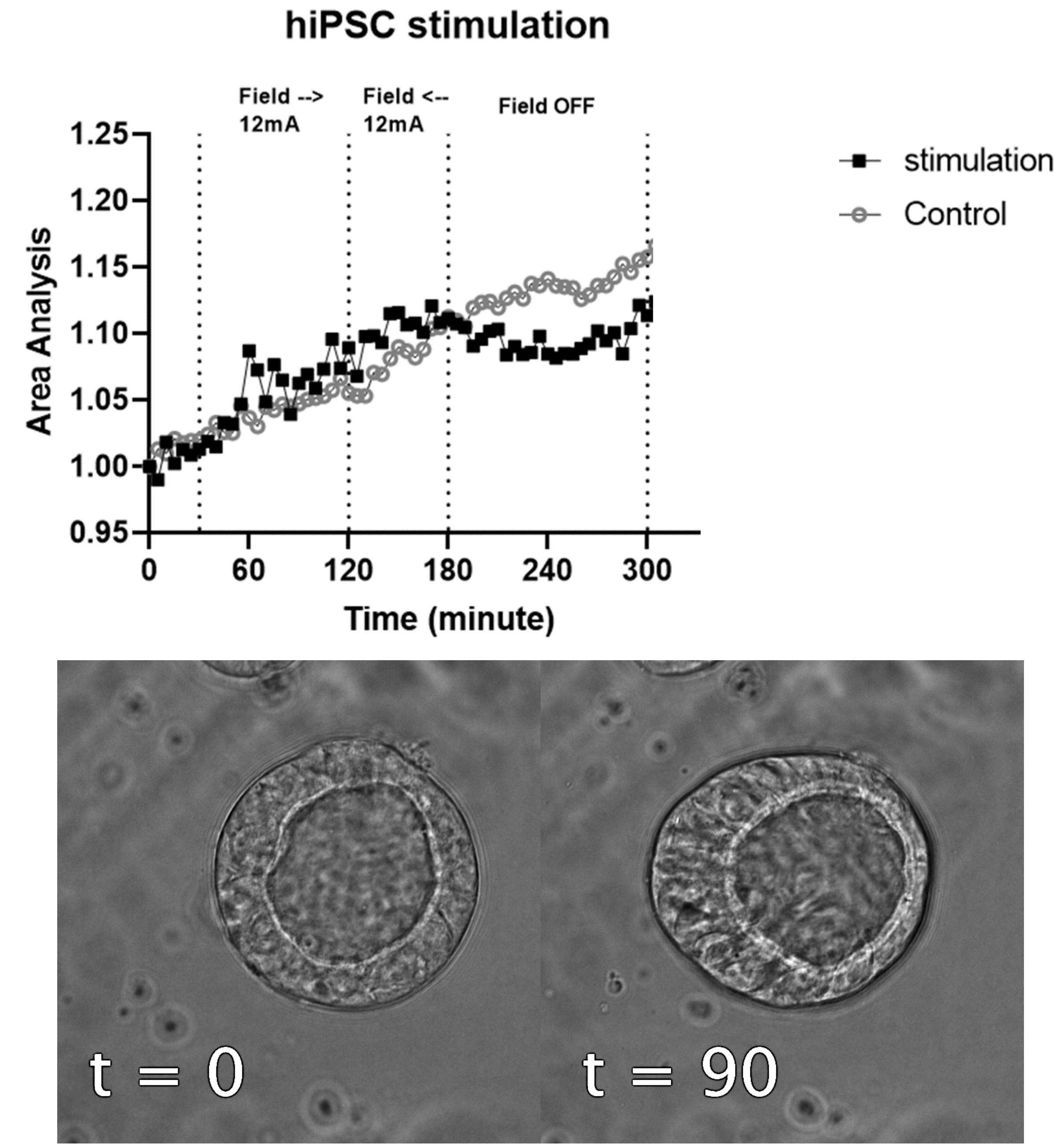

Supplementary Figure 18 Up: Comparison of inflation dynamics of hiPSC lumenized structures with (stimulation; N =1, n = 12) and without (control; N = 1, n = 47) electrical stimulation. Electrical stimulation does not seem to induce luminal inflation in hiPSC structures unlike in MDCK cysts.

Down: hiPSC spherical structures at initial timepoint (t = 0) and after 90 min of electrical stimulation at 12mA (t = 90 min). The structure does not show luminal inflation but clearly shows thinning-thickening asymmetry. Scale bar = 25um.

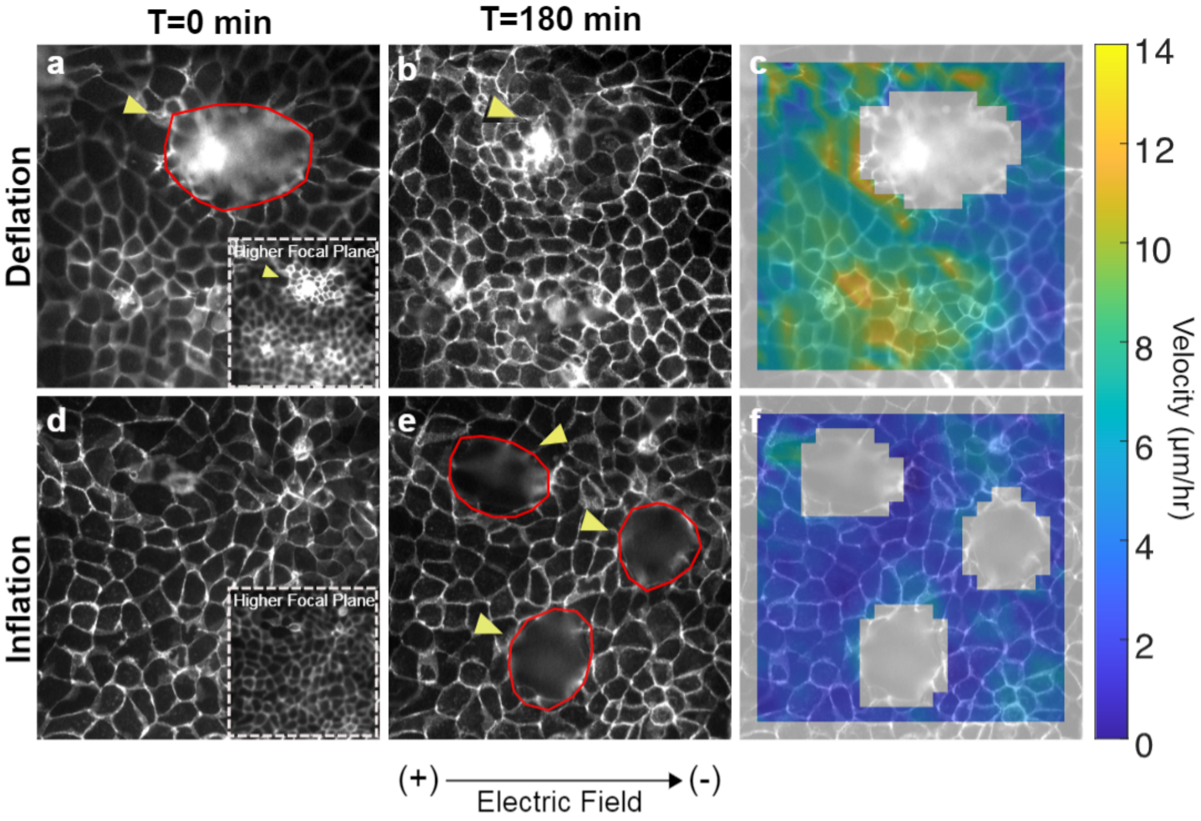

Supplementary Figure 19: **a.** MDCK dome (arrow) fully inflated, pre-stimulation. In fluorescence imaging, domes will appear as blurry or dark zones because they are high above the focal plane of the objective. Locally blurred regions of the image mean differences in 3D height (domes). To demonstrate this we show data from different focal planes (see inset). **b.** MDCK dome (arrow) fully deflated, post-stimulation. **c.** Heat map of velocity near the dome that deflates during stimulation, showing high migrational speeds in the region proximal to the dome. **d.** MDCK monolayer (*without* domes) pre-stimulation (see inset showing no in-focus cells out of plane). **e.** MDCK domes (arrows) that have inflated during stimulation. **f.** Heat map of velocities near the inflated domes, showing little to no movement proximal to the domes. Scale bar 25um.

Supplementary Note for "Bioelectric stimulation controls tissue shape and size"

Continuum model for cyst inflation. Here we derive a minimal continuum model to recapitulate the experimental observations of cyst inflation. In particular, we consider that inflation stems from hydrostatic and osmotic pressure differences across the cyst's epithelium. As a first approximation, we consider that the medium in which the cyst is embedded is a 1:1 electrolyte solution, with cations of valence $z_+ = |z| > 0$ and anions of valence $z_- = -|z| < 0$ (e.g., NaCl), and further assume that the variations of the electric potential with respect to an externally applied far-field electric potential ϕ_{sys} are small, i.e. $\phi \ll \phi_{\text{sys}}$.

An important quantity in electrokinetic phenomena is the Debye–Hückel (DH) length, which measures the thickness of a surface charge layer and a screening cloud of ions at such surface immersed in an electrolyte solution, usually referred to as diffuse double layer (DDL) or electrical double layer (EDL). The first layer corresponds to the surface charge, either positive or negative, and the second layer corresponds to ions attracted to the surface via Coulomb force, which electrically screen the first layer. Considering the estimates of parameter values in **Table S1**, we find that the DH length is $\ell_{\text{DH}} = \sqrt{\varepsilon\phi_c/(c_c|z|e)} \sim 10 - 20$ nm, where e is the elementary charge, ε is the permittivity of the medium, and c_c and ϕ_c are a characteristic ion concentration and electrostatic potential, respectively, which we take to be the background ion concentration of the electrolyte, $c_c = c^0_{\text{out}}$, and the applied voltage $\phi_c = \phi_{\text{sys}}$. Thus, such length is significantly smaller than the average radius of cysts in our experiments, $\ell_{\text{DH}} \ll R_{\text{init}}$, implying that a very thin DDL of positive and negative ions is formed at the basal surface of the cyst. We further assume that this layer is at quasi equilibrium, which allows us to obtain the electric potential from Poisson's equation and consider that the concentration of ions is Boltzmann distributed, i.e., $c_{\text{i,out}} = c^0_{\text{out}} \exp[-z_i e(\phi - \phi_{\text{sys}})/(k_B T)]$. Here the subscript i $= (\pm, -)$ denotes positive or negative ions. We have also considered that the background concentration of the electrolyte c^0_{out} is constant and is the same for all ion species at $\boldsymbol{r} \gg \ell_{\text{DH}}$, where we assume that the medium is neutral ($\boldsymbol{r}$ denotes the positional vector). Moreover, we follow Ref. [1] and linearize the chemical potentials, so that osmotic fluxes are linearly proportional to ion concentration. Assuming that the fluid inside the cyst is incompressible and $\phi \ll \phi_{\text{sys}}$, mass conservation equation and the linearized Poisson-Boltzmann equations read:

Lumen volume conservation:

$$\nabla \cdot (\boldsymbol{v} - j_{\text{flux}}) = 0, \ \text{ at } \ 0 \le r \le R$$
$$\text{with: } j_{\text{flux}} = \lambda_w(\Delta\Pi - \Delta p), \ \text{ at } r = R, \tag{S1a}$$

$$\text{Cell volume conservation: } \partial_t \left(h \int_S \mathrm{d}SR \right) = 0, \tag{S1b}$$

Poisson-Boltzmann eq. in the DDL:

$$\ell^2_{\text{DH}} \nabla^2(\phi - \phi_{\text{sys}}) = \phi - \phi_{\text{sys}}, \ \text{ at } R \le r \le \ell_{\text{DH}}, \tag{S1c}$$

Ion conservation in the lumen:

$$\int_V \mathrm{d}V\, \partial_t c_{\text{i,in}} = -\int_S \mathrm{d}S j_{\text{flux}} c_{\text{i,in}} + \int_S \mathrm{d}S j_{\text{active}} - \int_S \mathrm{d}S \lambda_{\text{ion}} \Big[c_{\text{i,in}} - c_{\text{i,out}}|_{r=R(t)} + \frac{z_i e^2 R(c_{\text{i,in}} + c_{\text{i,out}})}{12 k_B T C} \sum_i z_i \left(c_{\text{i,in}} - c_{\text{i,out}}|_{r=R(t)} \right) \Big], \tag{S1d}$$

where $R(\theta, \phi, t)$ and $h(\theta, \phi, t)$ denote the average radius and thickness of the cyst, respectively, $\boldsymbol{v} = (v_r, v_\theta, v_\phi)$ is the fluid velocity inside the cyst, ϕ is the electric potential in the medium where the cyst is embedded, and $\Delta\Pi$ and Δp are the osmotic and hydrostatic pressure differences across the epithelium, respectively. Here, λ_w and λ_{ion} are the water and ion permeability coefficients across the epithelium (assumed to be the same for cations and anions and uniform along the epithelium), respectively, and C is the capacitance of the epithelium. Moreover, R_f denotes a far-field system radius where a constant electric potential ϕ_{sys} is imposed. The term j_{active} describes the active pumping of ions by the epithelium of the cyst [1]. For simplicity we consider that the active pumping of ions is uniform along the surface and is not affected by the externally applied electric field. Nonetheless, a recent study suggests that the active pumping of ions may be affected locally by the trans-epithelial potential [2]. We follow previous works [1] and assume that electro-osmosis is negligible, i.e. a water flux driven by the trans-epithelial potential, compared to the permeation of water due to gradients of ion's chemical potential. Equation (S1b) assumes that the displacement along the thickness of the epithelium is uniform, and that cell proliferation is negligible during cyst swelling.

Equation (S1a) is complemented with the momentum conservation equation in the lumen and at the apical surface to determine the pressure p and velocity $\boldsymbol{v}$ inside the lumen, and with a kinematic condition relating the local velocity in the lumen with the cyst surface, i.e. $(\partial_t \boldsymbol{r}_{\text{surf}} - \boldsymbol{v}_{\text{surf}}) \cdot \boldsymbol{n} = 0$, where $\boldsymbol{n}$ is the unit normal vector to the surface, $\boldsymbol{r}_{\text{surf}}$ its position vector, and $\boldsymbol{v}_{\text{surf}}$ its velocity.

The osmotic pressure difference is given by the total ion concentration difference between the basal and apical surface, and the hydrostatic pressure difference is determined by the

Physical parameters	Definition	Range of values
λ_{w}	Water permeability constant	$10^{-9}-10^{-7}$ μm Pa^{-1} s^{-1} [1, 3, 4]
λ_{ion}	Ion permeability constant	$10^{-4}-1.7\times10^{-1}$ μm s^{-1} [1, 4, 5]
j_{active}	Active flux density	10^5-10^6 μm^{-2} s^{-1} [4, 6, 7]
q	Basal surface charge density	$1.1\times10^{-2}-1.4\times10^{-2}$ C m^{-2} [8, 9]
C	Capacitance of the Epithelium	10^{-2} pF μm^{-2} [10]
E_{cyst}	Epithelial Young's modulus	$5-30$ kPa [11, 12]
μ_{cyst}	Epithelial viscosity	10^5-10^6 Pa s [13, 14]
R_{init}	Initial radius of the cyst	$25-50$ μm
h_{init}	Initial thickness of the cyst	$5-10$ μm
Δp_{init}	Initial hydrostatic pressure	$20-300$ Pa [1, 4, 15, 16]
z	Valence	$\pm$ 1 (NaCl)
E_{gel}	Young's Modulus of Geltrex	30 ± 2 kPa
$c^0_{\text{i,out}}$	Outer background ion concentration	100 mM
$c^0_{\text{i,in}}$	Lumen initial ion concentration	100 mM
ϕ_{sys}	Far-field electric potential	1.1, 4.5, 6.5 V
ε	Permittivity of the outer medium	7×10^{-10} F m^{-1} $=74.5\epsilon_0$
r_{sys}	System size	3 cm
ℓ_{DH}	Debye-Hückel length (external field)	9, 18, 21.7 nm
$\ell^{\text{control}}_{\text{DH}}$	Debye-Hückel length (control)	1 nm
**Characteristic scales**		
$l_{\text{c}}=R_{\text{init}}$	Length	$25-50$ μm
$t_{\text{c}}=\dfrac{R_{\text{init}}}{\lambda_{\text{w}}k_{\text{B}}Tc^0_{\text{i,out}}}$	Time	$0.3-56$ h
$c_{\text{c}}=c^0_{\text{out}}$	Ion concentration	100 mM
$\phi_{\text{c}}=\phi_{\text{sys}}$	Electric potential	1.1, 4.5, 6.5 V
**Dimensionless parameters**		
$\tilde{R}_{\text{init}}=R_{\text{init}}/\ell_{\text{DH}}$	Initial size of the cyst/Debye-Hückel length	$10^3-5.5\times10^3$
$Q_{\text{i}}=q\ell_{\text{DH}}z_{\text{i}}e/(\varepsilon k_{\text{B}}T)$	DDL electric potential/Thermal electric potential	5.5, 11, 13.3
$\alpha=c^0_{\text{in}}/c^0_{\text{out}}$	Initial lumen ion concentration/Outer ion concentration	1
$\Lambda=\lambda_{\text{ion}}/(\lambda_{\text{w}}k_{\text{B}}Tc^0_{\text{in}})$	Ion permeability/Water permeability	$4\times10^{-5}-69$
$\beta=e^2R_{\text{init}}c^0_{\text{out}}/(k_{\text{B}}TC)$	Transepithelial potential strength	10^6
$\mathcal{E}_{\text{gel}}=E_{\text{gel}}/\Delta p_{\text{init}}$	Gel stiffness/Hydrostatic pressure	$15-300$
$\mathcal{E}_{\text{cyst}}=h_{\text{init}}E_{\text{cyst}}/(R_{\text{init}}\Delta p_{\text{init}})$	Epithelium stiffness/Hydrostatic pressure	0.25-480
$\tilde{\tau}=\tau/(\lambda_{\text{w}}k_{\text{B}}Tc^0_{\text{out}})$	Viscous relaxation time scale/Characteristic osmotic time scale	$1.7\times10^{-4}-1.9$
$\mathcal{P}=\Delta p_{\text{init}}/(k_{\text{B}}Tc^0_{\text{out}})$	Hydrostatic pressure/Osmotic pressure	$10^{-4}-10^{-2}$
$\tilde{j}_{\text{active}}=j_{\text{active}}/(\lambda_{\text{w}}k_{\text{B}}Tc^0_{\text{out}}c^0_{\text{in}})$	Active ion flux/Osmotic water flux	$6.7\times10^{-2}-6.7$

Table S1. Estimates and experimental measurements of the physical and dimensionless parameters of MDCK cysts used in the 1D model.

normal stress balance at the epithelium:

Osmotic pressure difference:

$$\Delta\Pi=k_{\text{B}}T\sum_i\left(c_{\text{i,in}}-c_{\text{i,out}}\right),\quad \text{at } r=R, \tag{S2a}$$

Hydrostatic pressure difference:

$$-\Delta p\boldsymbol{n}+\boldsymbol{\tau}\cdot\boldsymbol{n}=-\sigma h(\boldsymbol{\nabla}\cdot\boldsymbol{n})\boldsymbol{n}\quad \text{at } r=R, \tag{S2b}$$

where $\boldsymbol{\tau}$ is the viscous stress tensor of the fluid in the lumen, and σ denotes the wall stress of the epithelium, which we consider to be spatially uniform. Since cyst inflation is a slow process, we assume viscous stresses are small compared to the epithelium elastic resistance, and thus we disregard them [4]. We build upon previous work [15, 17, 18] and first assume that the cyst wall stress satisfies a purely elastic constitutive equation:

$$\partial_t\sigma=\frac{E_{\text{cyst}}}{R}\partial_t R, \tag{S3}$$

where E_{cyst} is the Young's modulus of the epithelium.

Regarding boundary conditions for the electric potential ϕ, we impose:

$$-\boldsymbol{n}\cdot\boldsymbol{\nabla}\phi=\frac{q}{\varepsilon},\quad \text{at } r=R, \tag{S4a}$$

$$\boldsymbol{e}_r\cdot\boldsymbol{\nabla}\phi=0,\quad \text{at } \boldsymbol{r}\gg\ell_{\text{DH}}, \tag{S4b}$$

where q is the charge surface density at the basal domain, assumed to be spatially uniform. Finally, as initial condition we impose that the cyst is perfectly spherical, i.e. $R(\theta,\phi,t=0)=R_{\text{init}}$.

To reduce the number of parameters of our model, we nondimensionalize the system of equations by scaling all vari-

ables with the following choice of characteristic scales:

$$\tilde{\boldsymbol{r}} = \frac{\boldsymbol{r}}{R_{\text{init}}}, \quad \tilde{\boldsymbol{v}} = \frac{\boldsymbol{v}}{\lambda_{\text{w}} k_{\text{B}} T c_{\text{out}}^0},$$
$$\tilde{t} = \frac{t}{R_{\text{init}}/(\lambda_{\text{w}} k_{\text{B}} T c_{\text{out}}^0)}, \quad \tilde{c}_{\text{i,in}} = \frac{c_{\text{i,in}}}{c_{\text{in}}^0},$$
$$\tilde{c}_{\text{i,out}} = \frac{c_{\text{i,out}}}{c_{\text{out}}^0}, \quad \tilde{\phi} = \frac{\phi}{k_{\text{B}} T/(|z|e)},$$
$$\Delta\tilde{p} = \frac{\Delta p}{\Delta p_{\text{init}}}, \quad \tilde{h} = \frac{h}{h_{\text{init}}}. \tag{S5}$$

where tildes denote dimensionless variables, and c_{in}^0 is the initial concentration of ions in the lumen, considered to be the same for cations and anions.

To elucidate the mechanisms underlying cyst inflation, we consider that the cyst maintains a perfectly spherical shape over time, i.e. $\tilde{r} = \tilde{R}(\tilde{t})$, and thus $\tilde{\boldsymbol{v}} = \tilde{v}_r(\tilde{r})\boldsymbol{e}_r$, where $\boldsymbol{e}_r$ is the radial unitary vector, $\tilde{\phi} = \tilde{\phi}(\tilde{r})$, and $\tilde{c}_{\text{i,out}} = \tilde{c}_{\text{i,out}}(\tilde{r})$. We first solve the 1D dimensionless version of the electric potential [Eq. (S1c)], with boundary conditions Eq. (S4), which yields

$$\tilde{\phi}_{\text{out}}(\tilde{r}) = \tilde{\phi}_{\text{sys}} + \frac{Q_{\text{i}} \tilde{R}^2 \tilde{R}_{\text{init}} \exp\left[(\tilde{R} - \tilde{r})\tilde{R}_{\text{init}}\right]}{\tilde{r}(1 + \tilde{R}\tilde{R}_{\text{init}})}, \tag{S6}$$

where:

$$\tilde{R}_{\text{init}} \equiv \frac{R_{\text{init}}}{\ell_{\text{DH}}} : \frac{\text{Initial cyst radius}}{\text{Debye-Hückel length}},$$
$$\tilde{\phi}_{\text{sys}} \equiv \frac{\phi_{\text{sys}}|z|e}{k_{\text{B}} T} : \frac{\text{Applied electric potential}}{\text{Thermal energy}},$$
$$Q_{\text{i}} \equiv \frac{q\ell_{\text{DH}} z_{\text{i}} e}{\varepsilon k_{\text{B}} T} : \frac{\text{Electric potential across the DDL}}{\text{Thermal electric potential}}. \tag{S7}$$

We can then obtain the dimensionless concentrations of ions outside the cyst as $\tilde{c}_{\text{i,out}} = \exp\left[-(\tilde{\phi}_{\text{out}} - \tilde{\phi}_{\text{sys}})\right]$. In particular, the concentration of ions at the cyst surface reads:

$$\tilde{c}_{\text{i,out}}(\tilde{r} = \tilde{R}) = \exp\left[-\frac{Q_{\text{i}} \tilde{R}\tilde{R}_{\text{init}}}{1 + \tilde{R}\tilde{R}_{\text{init}}}\right]. \tag{S8}$$

Solving Eq. (S1) and employing the boundary conditions Eq. (S9d) yields the following dimensionless system of equations:

$$\text{Cell volume conservation} : \frac{\mathrm{d}\tilde{h}}{\mathrm{d}\tilde{t}} = -\frac{2\tilde{h}}{\tilde{R}} \frac{\mathrm{d}\tilde{R}}{\mathrm{d}\tilde{t}}, \tag{S9a}$$

Lumen volume conservation :

$$\frac{\mathrm{d}\tilde{R}}{\mathrm{d}\tilde{t}} = \tilde{j}_{\text{flux}} = \left[\sum_{\text{i}} (\alpha\tilde{c}_{\text{i,in}} - \tilde{c}_{\text{i,out}}|_{\tilde{r}=\tilde{R}}) - \mathcal{P}\Delta\tilde{p}\right], \tag{S9b}$$

Ion species conservation in the lumen :

$$\frac{\tilde{R}}{3}\frac{\mathrm{d}\tilde{c}_{\text{i,in}}}{\mathrm{d}\tilde{t}} = -\tilde{j}_{\text{flux}}\tilde{c}_{\text{i,in}} + \tilde{j}_{\text{active}} - \Lambda\left[\alpha\tilde{c}_{\text{i,in}} - \tilde{c}_{\text{i,out}} + \beta\frac{\tilde{R}(\alpha\tilde{c}_{\text{i,in}} + \tilde{c}_{\text{i,out}})}{12}\sum_{\text{i}}(\tilde{c}_{\text{i,in}} - \tilde{c}_{\text{i,out}}|_{\tilde{r}=\tilde{R}})\right], \tag{S9c}$$

Epithelium elastic constitutive equation :

$$\frac{\mathrm{d}\Delta\tilde{p}}{\mathrm{d}\tilde{t}} = \frac{1}{\tilde{R}}\frac{\mathrm{d}\tilde{R}}{\mathrm{d}\tilde{t}}\left(\mathcal{E}_{\text{cyst}}\frac{2\tilde{h}}{\tilde{R}} - 3\Delta\tilde{p} + \mathcal{E}_{\text{gel}}\right), \tag{S9d}$$

with $\tilde{R}(\tilde{t} = 0) = \tilde{c}_{\text{i,in}}(0) = \Delta\tilde{p}(0) = \tilde{h}(0) = 1$ as initial conditions, where:

$$\alpha \equiv \frac{c_{\text{in}}^0}{c_{\text{out}}^0} : \frac{\text{Initial lumen ion concentration}}{\text{Initial outer ion concentration}}$$
$$\mathcal{P} \equiv \frac{\Delta P_{\text{init}}}{k_{\text{B}} T c_{\text{out}}^0} : \frac{\text{Hydrostatic pressure}}{\text{Osmotic pressure}},$$
$$\Lambda \equiv \frac{\lambda_{\text{ion}}}{\lambda_{\text{w}} k_{\text{B}} T c_{\text{in}}^0} : \frac{\text{Ion permeability}}{\text{Water permeability}},$$
$$\beta \equiv \frac{e^2 R_{\text{init}} c_{\text{out}}^0}{k_{\text{B}} T C} : \text{Transepithelial potential strength},$$
$$\mathcal{E}_{\text{cyst}} \equiv \frac{h_{\text{init}} E_{\text{cyst}}}{R_{\text{init}} \Delta p_{\text{init}}} : \frac{\text{Epithelium elasticity}}{\text{Initial hydrostatic pressure}},$$
$$\mathcal{E}_{\text{gel}} \equiv \frac{E_{\text{gel}}}{\Delta p_{\text{init}}} : \frac{\text{Gel stiffness}}{\text{Hydrostatic pressure}},$$
$$\tilde{j}_{\text{active}} \equiv \frac{j_{\text{active}}}{\lambda_{\text{w}} k_{\text{B}} T c_{\text{out}}^0 c_{\text{in}}^0} : \frac{\text{Active ion flux}}{\text{Osmotic water flux}}. \tag{S10}$$

The system of nonlinear first-order ordinary differential equations [Eqs. (S9a)-(S9d)] does not admit an analytical solution, thus we solve it numerically by standard methods.

Viscoelastic model. To test whether cyst material properties play a significant role on inflation dynamics, we modify the purely elastic constitutive equation [Eq. (S3)], and consider two of the simplest viscoelastic models, i.e.,

$$\text{Maxwell model} : \partial_t\sigma + \frac{\sigma}{\tau} = \frac{E_{\text{cyst}}}{R}\partial_t R, \tag{S11}$$

where τ is a viscous relaxation time scale stemming from $\tau = \mu_{\text{cyst}}/E_{\text{cyst}}$, where μ_{cyst} is the viscosity of the epithelium [13, 14], and

$$\text{Kelvin-Voigt model} : \sigma = E_{\text{cyst}}\left(\frac{R}{R_{\text{init}}} + \frac{\tau}{R}\partial_t R\right). \tag{S12}$$

The following rheological stress-strain relationships introduce an additional dimensionless parameter, $\tilde{\tau} = \tau/[R_{\text{init}}/(\lambda_{\text{w}} k_{\text{B}} T c^{0}_{\text{out}})]$, i.e., the ratio between the viscous relaxation time and the osmotic characteristic time scale (see **Table S1**).

Values of the dimensionless parameters used in the 1D simulations. To compare the experimental inflation curves with our theoretical model in Fig. 3 of the Main Text, we use the following values of the dimensionless parameters: $\tilde{R}_{\text{init}} = 10^3$ (and $\tilde{R}_{\text{init}} = 10^4$ for the control case), $Q_{\text{i}} = 3.75$, 4.25 ($Q_{\text{i}} = 0.5$ for the control case), $\alpha = 1$, $\Lambda = 0.4$, $\tilde{R}_{\text{in}} = 10^3$, $\mathcal{P} = 10^{-4}$, $\mathcal{E}_{\text{gel}} = 300$, $\mathcal{E}_{\text{cyst}} = 80$, $\beta = 10^6$, $\tilde{j}_{\text{active}} = 1.5$. These values of the dimensionless parameters correspond to the following values of the dimensional parameters: $\lambda_{\text{w}} = 10^{-9}$ μm Pa^{-1} s^{-1}, $\lambda_{\text{ion}} = 10^{-4}$ μm s^{-1}, $q = 1.1 \times 10^{-2}$ C m^{-2}, $C = 10^{-2}$ pF μm^{-2}, $E_{\text{cyst}} = 20$ kPa, $R_{\text{init}} = 50$ μm, $h_{\text{init}} = 5$ μm, $\Delta p_{\text{init}} = 25$ Pa, $E_{\text{gel}} = 30$ kPa, $c^0_{\text{i,in}} = c^0_{\text{i,out}} = 100$ mM, $\phi_{\text{sys}} = 1.1-6.5$ V, and $\epsilon = 7 \times 10^{-10}$ F m^{-1}. In particular, we use the active flux of ions to match the control inflation curve, which yields $j_{\text{active}} = 2.2 \times 10^4$ μm^{-2} s^{-1}. This values is one order of magnitude below the range of estimates and measurements in **Table S1**, while the remaining parameters agree well with the range of values. Using these values of the parameters, the characteristic scales are: $t_{\text{c}} = 56$ h, $l_{\text{c}} = 50$ μm, $c_{\text{c}} = 100$ mM, and $\phi_{\text{c}} = 1.1-6.5$ V.

To match the experimental curves when ion transport is inhibited (Fig. 3d of the Main Text), we use $\Lambda = 0.05$, which yields $\lambda_{\text{ion}} = 1.24 \times 10^{-5}$ μm s^{-1}.

Moreover, if a Maxwell material is considered, we always use $\tilde{\tau} = 2$, which is the upper limit value reported in **Table S1**, and $\Delta p_{\text{init}} = 50$ Pa. Finally, to capture the longer time relaxation dynamics of cysts, for simplicity we do not consider the coupling with electrokinetcs to capture inflation. Instead, we impose a constant osmotic pressure difference $\Delta\Pi$ over time (see Fig. 3 of the Main Text). Consequently, the agreement between experiments and theory during inflation is worse. Nonetheless, this choice simplifies the numerical calculation to test whether cysts relax in an elastic or a viscoelastic fashion. In particular, at the same time when stimulation is turned off in experiments, we decrease the osmotic pressure drastically, which could be a consequence of ion rearrangement or fluid leakage. Even though the osmotic pressure is still positive, it cannot overcome the high value of the normal stress induced by hydrostatic pressure. Inflation induces a large wall stress, thus imparting a large normal stress that drives cyst shrinkage.

Strengths and limitations of our theoretical 1D model. Given the inherent complexities of experiments and the simplifications made in our 1D theoretical model, can we still make quantitative predictions of cyst swelling? Our 1D model couples the mechanics of a thin elastic membrane (the epithelium), that is permeable to ions and water, with electrokinetics arising from an externally applied electric field. The model is able to capture the inflation dynamics exhibited by cysts in experiments, using reasonable values of the model parameters. Moreover, it also captures the inflation trends observed when ion transport an cytoskeletal inhibitors are used in experiments.

Nevertheless, our 1D model has limitations and cannot capture all experimental findings. For example, we find that, to match the experimental inflation curves, we have to impose a smaller value of the dimensionless parameter that quantifies the field strength, i.e., Q, than our estimate in **Table S1**. Similarly, to match the inflation dynamics of the control experiment, the value of the ion active flux j_{active} is smaller than our estimates in **Table S1**. Also, our 1D model cannot capture well the inflation curves of the experiment with cytoskeletal inhibitor by decreasing the Young's modulus.

Where do these discrepancies come from? Even though our model takes into account many of the complexities present in experiments, it disregards many others. For example, we neglect variations along the surface of the cyst. This approximation precludes us to predict any kind of asymmetry, as found in experiments. We also approximate the epithelium as a thin permeable purely elastic layer, and consider that the elastic modulus and permeability coefficients are uniform and decoupled from the osmotic fluxes and active ion fluxes. In particular, such simplifications may explain the discrepancies between theory and experiments found with cytoskeletal inhibitors.

Estimation of shear stress induced by electro-osmotic flow. To test whether the asymmetric deformation exhibited by MDCK acini is caused by the shear stresses at the basal surface induced by electro-osmotic flow, we first obtain the electro-osmotic slip velocity around a sphere. To this end, we consider volume and momentum conservation of the electro-osmotic flow assuming that inertia is negligible

$$\boldsymbol{\nabla} \cdot \boldsymbol{u} = 0, \tag{S13a}$$

$$\mu \boldsymbol{\nabla} \boldsymbol{u}^2 - \boldsymbol{\nabla} p = e(c_+ - c_-) \boldsymbol{\nabla} \phi, \tag{S13b}$$

where μ is the shear viscosity of the electrolyte, and $\boldsymbol{u}$ is the fluid velocity field. From Eq. (S13b) we can estimate the electro-osmotic velocity, i.e. $u_{\text{c}} \sim e c_0 \phi_{\text{sys}} R_{\text{init}}/\mu$, or equivalently, $u_{\text{c}} \sim \varepsilon E_0^2 R_{\text{init}}/\mu$. Considering $E_0 \sim 200$ V m^{-1}, $u_{\text{c}} \sim 1.7$ μm s^{-1}. Such velocity yields a Reynolds number of $Re \sim 0.02$, so we can safely neglect inertia.

We obtain an analytical solution for the electro-osmotic slip velocity. For the case of a sphere, the radial and azimuthal components of such velocity field read:

$$u_r = \frac{9 \varepsilon E_0^2 R_{\text{init}}^3 (R_{\text{init}}^2 - r^2)(1 + 3\cos 2\theta)}{16 \mu r^4}, \tag{S14a}$$

$$u_\theta = \frac{9 R_{\text{init}}^5 \varepsilon E_0^2 \sin 2\theta}{8 \mu r^2}. \tag{S14b}$$

The tangential component of the viscous shear stress is given

Physical parameters	Definition	Range of values
ε	Permittivity of the medium	7×10^{-10} F m^{-1} $= 74.5\epsilon_0$
D	Ion diffusivity in the outer medium	10^{-11}-10^{-9} m^2 s^{-1} [19]
D_{cyt}	Ion diffusivity in the cytoplasm	10^{-11}-10^{-9} m^2 s^{-1}
λ_{basal}	Basal surface ion permeability	$10^{-4} - 10^{-1}$ s^{-1} [1]
ζ	Basal surface zeta potential	20 mV [9]
E_∞	Applied electric field	0.1-10 V cm^{-2}
R_{init}	Cyst radius	25-50 μm
h_{init}	Epithelium thickness	$5 - 15$ μm
**Dimensionless parameters**		
$\tilde{R}_{\text{init}} \equiv R_{\text{init}}/\ell_{\text{DH}}$	Initial size of the cyst/Debye-Hückel length	$10^3 - 5.5 \times 10^3$
$\tilde{\zeta} \equiv \zeta/(k_{\text{B}}T/e)$	Basal surface zeta potential/Thermal electric potential	0.7
$\tilde{E}_\infty \equiv E_\infty/[k_{\text{B}}T/(eR_{\text{init}})]$	Applied electric field/Thermal electric field	$0.015 - 1.5$
$\Lambda \equiv \lambda_{\text{basal}}/(D/R_{\text{init}}^2)$	Basal surface ion permeability/Ion diffusivity	3×10^{-3}
$\mathcal{D} \equiv D_{\text{cyt}}/D$	Cytoplasm ion diffusivity/Outer medium ion diffusivity	0.01-100

Table S2. Estimates and experimental measurements of the physical and dimensionless parameters used in the 2D model.

by

$$\boldsymbol{e}_\theta \cdot \boldsymbol{\tau} \cdot \boldsymbol{e}_r = \tau_{r\theta} = \mu\left(\partial_r v_\theta - \frac{v_\theta}{r} + \frac{1}{r}\partial_\theta v_r\right) = \frac{9\varepsilon E_0^2 R_{\text{init}}^3(3r^2 - 8R_{\text{init}}^2)\sin 2\theta}{8r^5}. \tag{S15}$$

Hence, the shear stress associated with the flow right above the DDL is

$$\tau_{r\theta}|_{r=R_{\text{init}}} = \frac{45\varepsilon E_0^2}{8}\sin 2\theta. \tag{S16}$$

Using the estimate of the far-field electric field, i.e., $E_0 \sim 200$ V m^{-1}, yields a maximum shear stress of $\max(\tau_{r\theta}|_{r=R_{\text{init}}}) \sim 2 \times 10^{-4}$ Pa, or 2×10^{-3} dynes cm^{-2}. Such shear stress is too small to induce any significant deformation to the epithelium and significantly lower than shear forces reported to affect MDCK epithelial tissues [20]. Nonetheless, this shear stress needs to be corrected to account for the shear stress exerted by the electro-osmotic flow at the basal surface in the DDL. At the basal surface, the velocity is zero due to the no-slip condition, thus the velocity increases from $u = 0$ to $u_{\text{c}} \sim ec_0\phi_{\text{sys}}R_{\text{init}}/\mu$ outside the DDL. Although the increase in velocity is small, the thickness of the DDL is just a few nanometers, yielding a shear stress of $\tau_{\text{DDL}} \sim \mu u_{\text{c}}/\ell_{\text{DH}} \sim 0.1 - 1$ Pa, corresponding to $\ell_{\text{DH}} \sim 1 - 10$ nm, which is also too small to cause any deformation to the epithelium.

Continuum two-dimensional (2D) model for electrolyte transport. To describe the dynamics of an electrolyte solution surrounding a spherical cyst subject to an external electric field, we consider a two-dimensional axisymmetric continuum model that takes into account: the asymmetric electrostatic field outside the cyst and inside the epithelium, $\phi_{\text{out}}(\boldsymbol{r}, t)$ and $\phi_{\text{in}}(\boldsymbol{r}, t)$, respectively, which obey the Poisson's equation, and the conservation of positive, $c_+(\boldsymbol{r}, t)$, and negative, $c_-(\boldsymbol{r}, t)$, ion species, usually referred to as Poisson-Nernst-Planck equations [21–23]. The system of equations reads:

Electric potential :

$$\boldsymbol{\nabla}^2\phi_{\text{i}} = -\frac{(c_{+,\text{i}} - c_{-,\text{i}})e}{\varepsilon_{\text{i}}}, \tag{S17a}$$

Ion species conservation :

$$\partial_t c_{\pm,\text{i}} + \boldsymbol{\nabla}\cdot(c_{\pm,\text{i}}\boldsymbol{v}_{\pm,\text{i}}) = 0, \tag{S17b}$$

where $\text{i} = \{\text{in}, \text{out}\}$, $\boldsymbol{r}$ and t denote position and time, respectively, and $\boldsymbol{v}_{\pm,\text{i}}$ is the velocity of the ion species, i.e. $\boldsymbol{v}_{\pm,\text{i}} = \mp b_{\text{i}}e\boldsymbol{\nabla}\phi_{\text{i}} - k_{\text{B}}Tb_{\text{i}}\boldsymbol{\nabla}\ln c_{\pm,\text{i}}$. The first term accounts for electrostatic forcing of ions, and the second term represents diffusion of ions, where $b_{\text{i}} = D_{\text{i}}/(k_{\text{B}}T)$ is the mobility of ions and D_{i} the diffusivity in the outer medium. Regarding the boundary conditions, we impose no-flux at every surface for the ion concentration, a uniform zeta potential at the outer surface of the shell, and a far-field uniaxial electric field, i.e., $\boldsymbol{E} = E_\infty\boldsymbol{e}_z$.

To account for transport of ions across the basal surface, we impose the following permeation boundary conditions:

$$(\pm b_{\text{in}}e\boldsymbol{\nabla}\phi_{\text{in}} - D_{\text{in}}\boldsymbol{\nabla}c_{\pm,\text{in}})\cdot\boldsymbol{n} = -\lambda_{\text{basal}}(c_{\pm,\text{out}} - c_{\pm,\text{in}}), \tag{S18}$$

$$(\pm b_{\text{out}}e\boldsymbol{\nabla}\phi_{\text{out}} - D_{\text{out}}\boldsymbol{\nabla}c_{\pm,\text{out}})\cdot\boldsymbol{n} = \lambda_{\text{basal}}(c_{\pm,\text{out}} - c_{\pm,\text{in}}) \tag{S19}$$

at the basal surface, where λ_{basal} is the ion permeability constant of the basal surface, $c_{\pm,\text{in}}(\boldsymbol{r}, t)$ is the local density of ions in the epithelium, and $\boldsymbol{n}$ is the unit normal vector to the basal surface.

To perform full time-dependent numerical simulations, we non-dimensionalize Eqs. (S17) by choosing $\ell_{\text{c}} = R_{\text{init}}$, $t_{\text{c}} = R_{\text{init}}^2/D$, $\phi_{\text{c}} = k_{\text{B}}T/e$, $c_{\text{c}} = c_{\text{init}}$ as characteristic length, time, electric potential, and ion concentration scales, respectively.

The dimensionless version of Eqs. (S17) read,

$$\text{Outer electric potential :}$$
$$\nabla^2\tilde{\phi}_{\text{out}} = -\tilde{R}^2_{\text{init}}(\tilde{c}_{+,\text{out}} - \tilde{c}_{-,\text{out}}) \tag{S20a}$$
$$\text{Epithelial electric potential :}$$
$$\nabla^2\tilde{\phi}_{\text{in}} = -\frac{\tilde{R}^2_{\text{init}}}{\tilde{\varepsilon}}(\tilde{c}_{+,\text{in}} - \tilde{c}_{-,\text{in}}) \tag{S20b}$$
$$\text{Ion species conservation, outer medium :}$$
$$\partial_{\tilde{t}}\tilde{c}_{\pm,\text{out}} = \nabla^2\tilde{c}_{\pm,\text{out}} + \nabla\cdot(\tilde{c}_{\pm,\text{out}}\nabla\tilde{\phi}_{\text{out}}), \tag{S20c}$$
$$\text{Ion species conservation, epithelium :}$$
$$\partial_{\tilde{t}}\tilde{c}_{\pm,\text{in}} = \mathcal{D}\nabla^2\tilde{c}_{\pm,\text{in}} + \nabla\cdot(\tilde{c}_{\pm,\text{in}}\nabla\tilde{\phi}_{\text{in}}), \tag{S20d}$$

with the following boundary conditions for the dimensionless electric potential $\tilde{\phi}$ at the basal surface and $|\tilde{\boldsymbol{r}}| \gg 1$:

$$\text{Basal zeta potential : } \tilde{\phi}_{\text{i}} = \tilde{\zeta}, \tag{S21a}$$
$$\text{Far-field electric field : } -\nabla\tilde{\phi}_{\text{out}}\cdot\boldsymbol{e}_z = \tilde{E}_\infty, \tag{S21b}$$
$$\text{Basal ion permeation :}$$
$$(\pm\nabla\phi_{\text{in}} - \mathcal{D}\nabla\tilde{c}_{\pm,\text{in}})\cdot\boldsymbol{n} = -\Lambda(\tilde{c}_{\pm,\text{out}} - \tilde{c}_{\pm,\text{in}}),$$
$$(\pm\nabla\phi_{\text{out}} - \nabla\tilde{c}_{\pm,\text{out}})\cdot\boldsymbol{n} = \Lambda(\tilde{c}_{\pm,\text{out}} - \tilde{c}_{\pm,\text{in}}). \tag{S21c}$$

The dimensionless parameters that emerge in Eqs. (S20) are:

$$\tilde{\zeta} \equiv \frac{\zeta}{k_{\text{B}}T/e} : \frac{\text{Basal surface zeta potential}}{\text{Thermal electric potential}},$$
$$\tilde{E}_\infty \equiv \frac{E_\infty}{k_{\text{B}}T/(eR_{\text{init}})} : \frac{\text{Applied electric field}}{\text{Thermal electric field}},$$
$$\Lambda \equiv \frac{\lambda_{\text{basal}}}{D/R^2_{\text{init}}} : \frac{\text{Basal surface ion permeability}}{\text{Ion diffusivity}},$$
$$\mathcal{D} \equiv \frac{D_{\text{cyt}}}{D} : \frac{\text{Cytoplasm ion diffusivity}}{\text{Outer medium ion diffusivity}},$$
$$\tilde{\varepsilon} \equiv \frac{\varepsilon_{\text{cyt}}}{\varepsilon} : \frac{\text{Cytoplasm permittivity}}{\text{Outer medium permittivity}}. \tag{S22}$$

2D numerical simulations. We perform full time-dependent numerical simulations using the finite-element method, which involves writing the equations in weak form by means of the integral scalar product using test functions $\tilde{\varphi}_\phi$ and $\tilde{\varphi}_c$ for the electric potential $\phi(\tilde{\boldsymbol{r}},\tilde{t})$ and ion concentration $\tilde{c}_\pm(\tilde{\boldsymbol{r}},\tilde{t})$ fields, respectively. Using Green identities, we obtain an integral bilinear system of equations for the variables and their test functions:

$$-\int_{\mathcal{V}} \mathrm{d}\mathcal{V}\,\nabla\tilde{\phi}\cdot\nabla\tilde{\varphi}_\phi + \int_{\mathcal{V}} \mathrm{d}\mathcal{V}\,\tilde{R}^2_{\text{init}}(\tilde{c}_+ - \tilde{c}_-)\,\tilde{\varphi}_\phi$$
$$-\,\mathrm{d}\mathcal{C}\,\nabla\tilde{\phi}\cdot\boldsymbol{n}\,\tilde{\varphi}_c = 0, \tag{S23a}$$
$$\int_{\mathcal{V}} \mathrm{d}\mathcal{V}\,\partial_{\tilde{t}}\tilde{c}_\pm\,\tilde{\varphi}_c + \int_{\mathcal{V}} \mathrm{d}\mathcal{V}\,(\nabla\tilde{c}_\pm + \tilde{c}_\pm\nabla\tilde{\phi})\cdot\nabla\tilde{\varphi}_c$$
$$-\int_{\mathcal{C}} \mathrm{d}\mathcal{C}(\nabla\tilde{c}_\pm + \tilde{c}_\pm\nabla\tilde{\phi})\cdot\boldsymbol{n}\,\tilde{\varphi}_c = 0. \tag{S23b}$$

Here, $\boldsymbol{n}$ is the outer unit normal of the boundaries $\mathcal{C}$ of the 2D domain $\mathcal{V}$, and $\mathrm{d}\mathcal{V}$ and $\mathrm{d}\mathcal{C}$ are the surface and line elements, respectively. To ensure numerical stability, the equations are discretized in space using second-order Lagrange polynomials and triangular elements for the fields, and evolved in time through a 4th-order variable-step backward differentiation formula method. The relative tolerance of the nonlinear method is always set below 10^{-6}.

Values of the dimensionless parameters used in the 2D simulations. To obtain Figs. 3c and 5a of the Main Text, we used the following values of the dimensionless parameters: $\tilde{\zeta} = 0.7$, $\tilde{E}_\infty = 0.015 - 1.5$, $\mathcal{D} = 1$, $\tilde{\varepsilon} = 0.1$, $\Lambda = 0.1$, which correspond to $R_{\text{init}} = 38\ \mu$m, $E_\infty = 0.1 - 10$ V cm^{-1}, $\zeta = 20$ mV, $\varepsilon = 7\times10^{-10}$ F m^{-1}, $\varepsilon_{\text{cyt}} = 7\times10^{-10}$ F m^{-1}, and $D = D_{\text{cyt}} = 10^{-10}\ \mu$m^2 s^{-1}. Such values are within the ranges of parameter estimates reported in **Table S2**. Fully resolving the DDL and the outer solution at high values of the applied electric field E_∞ is numerically challenging, as the thickness of the DDL becomes extremely small compared to the sizes of the cyst and the domain, implying a disparate separation of length scales. To circumvent this issue, in Fig. 3c of the Main Text, we relax the thickness of the DDL and perform simulations considering $\tilde{R}_{\text{init}} = 10$.

[1] A. Torres-Sánchez, M. K. Winter, and G. Salbreux, Cells Dev. **168**, 203724 (2021).
[2] Y. Li and S. Sun, bioRxiv pp. 2023–07 (2023).
[3] K. Olbrich, W. Rawicz, D. Needham, and E. Evans, Biophys. J. **79**, 321 (2000).
[4] E. Latorre, S. Kale, L. Casares, M. Gómez-González, M. Uroz, L. Valon, R. V. Nair, E. Garreta, N. Montserrat, A. Del Campo, et al., Nature **563**, 203 (2018).
[5] S. Fernández-Castelo, J. J. Bolívar, R. López-Vancell, G. Beaty, and M. Cereijido, *Ion Transport in MDCK Cells* (Springer US, Boston, MA, 1985), pp. 37–50, ISBN 978-1-4684-4814-6.
[6] M. Cereijido, J. Ehrenfeld, S. Fernandez-Castelo, and I. Meza, Ann. N. Y. Acad. Sci. **372**, 422 (1981).
[7] N. Simmons, J. Membr. Biol. **59**, 105 (1981).
[8] O. V. Bondar, D. Saifullina, I. Shakhmaeva, I. Mavlyutova, and T. Abdullin, Acta Nat. **4**, 78 (2012).
[9] A. Sarkar, B. M. Kobylkevich, D. M. Graham, and M. A. Messerli, J. Theor. Biol. **478**, 58 (2019).
[10] J. Wegener, M. Sieber, and H.-J. Galla, J. Biochem. Biophys. Methods **32**, 151 (1996).
[11] R. A. Foty, G. Forgacs, C. M. Pfleger, and M. S. Steinberg, Phys. Rev. Lett. **72**, 2298 (1994).
[12] K. Schulze, S. Zehnder, J. Urueña, T. Bhattacharjee, W. Sawyer, and T. Angelini, J. Biomech. **53**, 210 (2017).
[13] P. Marmottant, A. Mgharbel, J. Käfer, B. Audren, J.-P. Rieu, J.-C. Vial, B. Van Der Sanden, A. Marée, F. Graner, and H. Delanoë-Ayari, Proc. Natl. Acad. Sci. U.S.A. **106**, 17271 (2009).
[14] K. Guevorkian, M.-J. Colbert, M. Durth, S. Dufour, and F. Brochard-Wyart, Phys. Rev. Lett. **104**, 218101 (2010).
[15] C. J. Chan, M. Costanzo, T. Ruiz-Herrero, G. Mönke, R. J. Petrie, M. Bergert, A. Diz-Munoz, L. Mahadevan, and T. Hiiragi, Nature **571**, 112 (2019).
[16] V. Narayanan, L. Schappell, C. Mayer, A. Duke, T. Armiger,

P. Arsenovic, A. Mohan, K. Dahl, J. Gleghorn, and D. Conway, Curr. Biol. **30**, 624 (2020).

[17] T. Ruiz-Herrero, K. Alessandri, B. V. Gurchenkov, P. Nassoy, and L. Mahadevan, Development **144**, 4422 (2017).

[18] M. Bagnat, B. Daga, and S. Di Talia, Annu. Rev. Cell Dev. Biol. **38** (2022).

[19] I. Eydelnant, B. Betty Li, and A. Wheeler, Nat. Commun. **5**, 1 (2014).

[20] J. Heo, F. Sachs, J. Wang, and S. Hua, Cell. Physiol. Biochem. **30**, 395 (2012).

[21] M. Z. Bazant and T. M. Squires, Phys. Rev. Lett. **92**, 066101 (2004).

[22] T. M. Squires and M. Z. Bazant, J. Fluid Mech. **509**, 217 (2004).

[23] S. M. Davidson, M. B. Andersen, and A. Mani, Phys. Rev. Lett. **112**, 128302 (2014).
