# Supplementary material for: Bioelectric stimulation controls tissue shape and size
Source: Nat Commun. 2024 Apr 5;15:2938. doi: 10.1038/s41467-024-47079-w (PMC10997591; doi:10.1038/s41467-024-47079-w)
Supplement: Supplementary file 2 — Reporting Summary [file 41467_2024_47079_MOESM2_ESM.pdf]

nature portfolio

Corresponding author(s): Dr. Daniel Cohen

Last updated by author(s): Feb 15, 2024

Reporting Summary

Nature Portfolio wishes to improve the reproducibility of the work that we publish. This form provides structure for consistency and transparency in reporting. For further information on Nature Portfolio policies, see our Editorial Policies and the Editorial Policy Checklist.

Statistics

For all statistical analyses, confirm that the following items are present in the figure legend, table legend, main text, or Methods section.

n/a	Confirmed	
☐	☒	The exact sample size (*n*) for each experimental group/condition, given as a discrete number and unit of measurement
☐	☒	A statement on whether measurements were taken from distinct samples or whether the same sample was measured repeatedly
☐	☒	The statistical test(s) used AND whether they are one- or two-sided<br>*Only common tests should be described solely by name; describe more complex techniques in the Methods section.*
☐	☒	A description of all covariates tested
☐	☒	A description of any assumptions or corrections, such as tests of normality and adjustment for multiple comparisons
☐	☒	A full description of the statistical parameters including central tendency (e.g. means) or other basic estimates (e.g. regression coefficient) AND variation (e.g. standard deviation) or associated estimates of uncertainty (e.g. confidence intervals)
☐	☒	For null hypothesis testing, the test statistic (e.g. *F*, *t*, *r*) with confidence intervals, effect sizes, degrees of freedom and *P* value noted<br>*Give P values as exact values whenever suitable.*
☒	☐	For Bayesian analysis, information on the choice of priors and Markov chain Monte Carlo settings
☒	☐	For hierarchical and complex designs, identification of the appropriate level for tests and full reporting of outcomes
☒	☐	Estimates of effect sizes (e.g. Cohen's *d*, Pearson's *r*), indicating how they were calculated

Our web collection on statistics for biologists contains articles on many of the points above.

Software and code

Policy information about availability of computer code

Data collection	Slidebook capture software (Intelligent Imaging Innovations, 3i, version: 7.2.4), NIS Elements software (Nikon, version: X.4.1), and Micro-Manager software (version: 2.3.7) was used to collect microscopy data. Custom code developed for the study's image analysis and numerical simulations can be found on GitHub (DOI: 10.5281/zenodo.10659296).
Data analysis	MATLAB (version: R2021a) ImageJ (FIJI, version: 2.14.0) was used for image analysis and quantification. GraphPad PRISM (version: 10) was used for data visualization and statistial tests. Computational analysis was done using MATLAB software (version: R2021a) and COMSOL (version 5.4).

For manuscripts utilizing custom algorithms or software that are central to the research but not yet described in published literature, software must be made available to editors and reviewers. We strongly encourage code deposition in a community repository (e.g. GitHub). See the Nature Portfolio guidelines for submitting code & software for further information.

Data

Policy information about availability of data

All manuscripts must include a data availability statement. This statement should provide the following information, where applicable:

- Accession codes, unique identifiers, or web links for publicly available datasets
- A description of any restrictions on data availability
- For clinical datasets or third party data, please ensure that the statement adheres to our policy

The inflation quantification data generated in this study are provided in the Supplementary Information/Source Data file. Source data are provided in this paper. The

microscopy image data used in this study are available in the zenodo database under accession code 7348438 (DOI: 10.5281/zenodo.7348438).

Research involving human participants, their data, or biological material

Policy information about studies with human participants or human data. See also policy information about sex, gender (identity/presentation), and sexual orientation and race, ethnicity and racism.

Reporting on sex and gender	N/A
Reporting on race, ethnicity, or other socially relevant groupings	N/A
Population characteristics	N/A
Recruitment	N/A
Ethics oversight	N/A

Note that full information on the approval of the study protocol must also be provided in the manuscript.

Field-specific reporting

Please select the one below that is the best fit for your research. If you are not sure, read the appropriate sections before making your selection.

- [x] Life sciences
- [ ] Behavioural & social sciences
- [ ] Ecological, evolutionary & environmental sciences

For a reference copy of the document with all sections, see nature.com/documents/nr-reporting-summary-flat.pdf

Life sciences study design

All studies must disclose on these points even when the disclosure is negative.

Sample size	No statistical methods were used to predetermine sample size. Each experiment was repeated 2-6 times to ensure that the observed phenotype was repeatable, and experimental replicate sizes were determined based on the current standard used for publications using epithelial model systems(Xi 2017 Nat. Comm (10.1038/s41467-017-01390-x), Shim 2021 PNAS (10.1073/pnas.2101352118)). Each experiment yielded 30-50 cyst sample sizes that could be used for data analysis and was sufficient for statistical analysis. We chose to use the pairwise parametric t-test with Welch's correction, which does not depend on a minimal sample size and is robust for large sample sizes, skewed distributions, and for comparing conditions with unequal sample sizes.
Data exclusions	MDCK cyst samples were excluded from area analaysis if the cyst did not have the characteristic spherical morphology or exhibited behavior that would prevent fair analysis (ex. merging with a neighboring cyst). Images with external non-cellular artefacts (debris, smudges on substrate, etc.) that overshadowed the cyst were filtered using a predetermined algorithm.
Replication	Experiments presented in the main figures were repeated 2-6 times across multiple days to ensure the results were reliable and repeatable.
Randomization	Cyst samples were chosen solely on whether they showed characteristic spherical morphology and could be reliably analyzed (see data exclusion).
Blinding	Researchers were not blinded for data collection as all cysts per experimental condition which had the characteristic spherical morphology were collected systematically to ensure unbiased representation. Additionally the quantified phenotype, the size of cysts under electrical stimulation and pharmaceutical treatment, was not readily identifiable by the human eye. Computational analysis was not performed blinded as computational algorithms utilized in our analysis required access to all relevant data inputs, and necessitated continuous iteration and refinement throughout the study.

Reporting for specific materials, systems and methods

We require information from authors about some types of materials, experimental systems and methods used in many studies. Here, indicate whether each material, system or method listed is relevant to your study. If you are not sure if a list item applies to your research, read the appropriate section before selecting a response.

Materials & experimental systems

n/a	Involved in the study
☐	☒ Antibodies
☐	☒ Eukaryotic cell lines
☒	☐ Palaeontology and archaeology
☒	☐ Animals and other organisms
☒	☐ Clinical data
☒	☐ Dual use research of concern
☒	☐ Plants

Methods

n/a	Involved in the study
☒	☐ ChIP-seq
☒	☐ Flow cytometry
☒	☐ MRI-based neuroimaging

Antibodies

Antibodies used	For ZO-1 staining, primary antibody anti ZO-1 antibody R40.76 (Santa Cruz Biotechnology, SC-33725) was used at 1:100 dilution with secondary antibody Goat anti-Rat IgG (H+L). Cross-Adsorbed Secondary Antibody, Alexa Fluor™ 647 (Invitrogen, A21247) was used at 1:500 dilution. For podocalyxin staining, anti-Podocalyxin (GP135) antibody, clone 3F2:D8 (Sigma-Aldrich, MABS1327) was used at 1:1000 dilution with secondary antibody Rabbit anti-Mouse IgG (H+L). Cross-Adsorbed Secondary Antibody, Alexa Fluor™ 488 (Invitrogen, A11059) was used at 1:500 dilution.
Validation	Anti ZO-1 antibody R40.76 was validated using western blot by the manufacturer in Caco-2 and mouse lungs cell lysates (https://www.scbt.com/p/zo-1-antibody-r40-76) and immunofluorescence in MDCK cells in a previous publication (Cong, W. et al, Curr. Biol., 20, 1408–1414 (2010)). Anti-Podocalyxin (GP135) was validated using western blotting in MDCK cell lysates by the manufacturer (Quality control: https://www.sigmaaldrich.com/US/en/product/mm/mabs1327).

Eukaryotic cell lines

Policy information about cell lines and Sex and Gender in Research

Cell line source(s)	WT MDCK-II and Ecad:dsRed MDCK-II: Shared from the Nelson Lab, Stanford University<br><br>Actin-GFP human induced pluripotent stem cells (hiPSC): Purchased from Allen Institute for Cell Science under the listing AICS-0016-184:WTC-mEGFP-ACTB-cl184(mono-allelictag).<br><br>Intestinal Stem Cell (ISC) organoids from CD-1 mouse: Shared by the Posfai Lab, Princeton University. Ethical approval was not required for the isolation of intestinal stem cells. The mouse small intestine for intestinal stem cell isolation was shared by researchers in the Posfai lab, collected from 1 mouse carcass (sex: female, strain: CD-1, age: 6 months) that had been sacrificed for embryo collection under another study. As the mouse small intestine were collected from an already sacrificed animal and shared with the authors, the collection did not involve the use of experimental animals. Additionally, the sex of the animal was not a factor in the isolation process, as intestinal stem cells are not influenced by the gender of the animal.
Authentication	None of the cells used were authenticated.
Mycoplasma contamination	All cells were regularly tested negative for mycoplasma with the most recent negative test from November 2023.
Commonly misidentified lines (See ICLAC register)	No commonly misidentified lines were used in this study.

Plants

Seed stocks	N/A
Novel plant genotypes	N/A
Authentication	N/A
