# Supplementary material for: Bioelectric stimulation controls tissue shape and size
Source: Nat Commun. 2024 Apr 5;15:2938. doi: 10.1038/s41467-024-47079-w (PMC10997591; doi:10.1038/s41467-024-47079-w)
Supplement: Supplementary file 3 — Description of Additional Supplementary Files [file 41467_2024_47079_MOESM3_ESM.pdf]

Supplementary Video 1
40x confocal image video of MDCK cyst inflating isotropically under medium current stimulation. Green: membrane dye (Memglow 488); cyan: nuclei (Hoechst 33342). Electrical stimulation indicated with “Field ->”. 3h stimulation on. 10 min/frame. Scale bar = 50um.

Supplementary Video 2
40x confocal image video of MDCK cyst undergoing structural asymmetry as it inflates under medium current stimulation. Green: membrane dye (Memglow 488); cyan: nuclei (Hoechst 33342). Electrical stimulation indicated with “Field ->”. 3h stimulation on. 10 min/frame. Scale bar = 50um.

Supplementary Video 3
10x transmitted light image video and binarization of inflating MDCK cyst under medium current stimulation. Electrical stimulation indicated with “Field ->”. 1h stimulation off, 4h stimulation on, 3h stimulation off. 10 min/frame. Scale bar = 50um.

Supplementary Video 4
10x transmitted light (left; grey) and EthD-1 live imaging (right; red) for cellular viability check. Structure inflates steadily with electrical stimulation. Electrical stimulation indicated with “Field ->”. 1h stimulation off, 4h stimulation on, 3h stimulation off. 10 min/frame. Scale bar = 50um.

Supplementary Video 5
10x transmitted light (left; grey) and EthD-1 live imaging (right; red) for cellular viability check. Structure inflates rapidly, ruptures, and deflates in the presence of electrical stimulation. Electrical stimulation indicated with “Field ->”. 1h stimulation off, 4h stimulation on, 3h stimulation off. 10 min/frame. Scale bar = 50um.

Supplementary Video 6
40x confocal image video of MDCK cyst treated with 100uM blebbistatin inflating under medium current stimulation. Green: membrane dye (Memglow 488); cyan: nuclei (Hoechst 33342). Electrical stimulation indicated with “Field ->”. 2.5min/ frame. Scale bar = 50um.

Supplementary Video 7
40x confocal image video of inflating RFP-Ecadherin MDCK cyst under oscillating directional field at 1.1mA/mm^2 stimulation. Direction of electrical stimulation indicated with “Field ->” and “Field <-”. 15 min control, 1h stimulation with field going to the right, 1h stimulation with field going to the left, 1h stimulation off. 15 min/frame. Scale bar = 50um.

Supplementary Video 8
40x confocal image video of MDCK cyst undergoing structural asymmetry but exhibiting minimal luminal inflation under medium current stimulation, suggesting inflation and asymmetry are uncoupled. Green: membrane dye (Memglow 488); cyan: nuclei (Hoechst 33342). Electrical stimulation indicated with “Field ->”. 3h stimulation on. 10 min/frame. Scale bar = 50um.

Supplementary Video 9
40x confocal image video of lumenized hiPSC structures forming asymmetry under high current stimulation (12mA). Green: GFP-actin; cyan: nuclei (Hoechst 33342). Electrical stimulation indicated with “Field ->”. 2.5min/ frame. Scale bar = 50um.

Supplementary Video 10
40x confocal image video of MDCK cyst inflating under electrical stimulation. Individual nuclei were tracked with TrackMate function in ImageJ to confirm that cells migrated along the electrical field vector lines that wrap around the cyst as shown in computational results. Migration tracks are

temporally color-coded and proceed along the parula colormap on the lower left (blue - > red). Electrical stimulation indicated with “Field ->”. 10min/ frame. Scale bar = 20um.

Supplementary Video 11
5x transmitted light image video of MDCK monolayers treated with 50uM LY294002 migrating towards the anode (non-treated monolayers migrate towards the cathode). Electrical stimulation indicated with “Field ->”. 10min/ frame. Scale bar = 500um.

Supplementary Video 12
40x confocal image video of MDCK cyst treated with 50uM LY294002 inflating under medium current stimulation. Fluorescence: membrane (MemGlow 488). Electrical stimulation indicated with “Field ->”. 10min/ frame. Scale bar = 20um.

Supplementary Video 13
10x phase video of RFP-E-cadherin MDCK domes with no treatment deflating with electrical stimulation. Note the high migrational speeds near the dome. Field directed towards the right, 9mA. 10 min/frame. Scale bar = 200um.

Supplementary Video 14
40x confocal cross-section of an RFPE-cadherin MDCK dome inflating with electrical stimulation. Note the low migrational speeds near the dome. 10 min/frame. Field directed towards the right, 9mA. Scale bar = 25um.

Supplementary Video 15
Crops of 10x phase videos of RFP-Ecadherin MDCK domes treated with 25uM LY294002 inflating with electrical stimulation. Note the low migrational speeds near the dome. Field directed towards the right, 9mA. Electrical stimulation indicated with “Field ->”. 5 min/frame. Scale bar = 250um.

Supplementary Video 16
20x transmitted light image video of inflating mouse intestinal stem cell organoid under medium current stimulation. Left crypt inflates significantly with stimulation. Electrical stimulation indicated with “Field ->”. 30 min stimulation off, 2h 30 min stimulation on, 1h stimulation off. 1 min/frame. Scale bar = 100um.

Supplementary Video 17
20x transmitted light image video of inflating mouse intestinal stem cell organoid under medium current stimulation. Left crypt inflates significantly with stimulation. Electrical stimulation indicated with “Field ->”. 30 min stimulation off, 2h 30 min stimulation on, 1h stimulation off. 1 min/frame. Scale bar = 100um.
